# A Looming Spatial Localization Neural Network Inspired by MLG1 Neurons in the Crab *Neohelice*

**DOI:** 10.3389/fnins.2021.787256

**Published:** 2022-01-21

**Authors:** Hao Luan, Qinbing Fu, Yicheng Zhang, Mu Hua, Shengyong Chen, Shigang Yue

**Affiliations:** ^1^School of Computer Science and Engineering, Tianjin University of Technology, Tianjin, China; ^2^Machine Life and Intelligence Research Centre, School of Mathematics and Information Science, Guangzhou University, Guangzhou, China; ^3^Computational Intelligence Laboratory (CIL), School of Computer Science, University of Lincoln, Lincoln, United Kingdom

**Keywords:** motion-sensitive neuron, MLG1, spatial localization, crab, visual motion perception

## Abstract

Similar to most visual animals, the crab *Neohelice granulata* relies predominantly on visual information to escape from predators, to track prey and for selecting mates. It, therefore, needs specialized neurons to process visual information and determine the spatial location of looming objects. In the crab *Neohelice granulata*, the Monostratified Lobula Giant type1 (MLG1) neurons have been found to manifest looming sensitivity with finely tuned capabilities of encoding spatial location information. MLG1s neuronal ensemble can not only perceive the location of a looming stimulus, but are also thought to be able to influence the direction of movement continuously, for example, escaping from a threatening, looming target in relation to its position. Such specific characteristics make the MLG1s unique compared to normal looming detection neurons in invertebrates which can not localize spatial looming. Modeling the MLG1s ensemble is not only critical for elucidating the mechanisms underlying the functionality of such neural circuits, but also important for developing new autonomous, efficient, directionally reactive collision avoidance systems for robots and vehicles. However, little computational modeling has been done for implementing looming spatial localization analogous to the specific functionality of MLG1s ensemble. To bridge this gap, we propose a model of MLG1s and their pre-synaptic visual neural network to detect the spatial location of looming objects. The model consists of 16 homogeneous sectors arranged in a circular field inspired by the natural arrangement of 16 MLG1s' receptive fields to encode and convey spatial information concerning looming objects with dynamic expanding edges in different locations of the visual field. Responses of the proposed model to systematic real-world visual stimuli match many of the biological characteristics of MLG1 neurons. The systematic experiments demonstrate that our proposed MLG1s model works effectively and robustly to perceive and localize looming information, which could be a promising candidate for intelligent machines interacting within dynamic environments free of collision. This study also sheds light upon a new type of neuromorphic visual sensor strategy that can extract looming objects with locational information in a quick and reliable manner.

## 1. Introduction

How to improve collision detection and avoidance remains a critical challenge for self-navigating robots, vehicles, and unmanned aerial vehicles (UAVs). Evasion strategies might be improved if the mobile machines can obtain and react to the spatial positions of continually approaching objects. Current schemes, such as radar, infra-red, laser, etc., or combinations of these, are acceptable but far from perfection in terms of their reliability, systematic complexity, or energy consumption. Amongst those, visual-based sensors are more ubiquitous and often accompanied by a compact hardware system. In addition, vision sensors have the advantage of being non-invasive, and are readily accepted for numerous application scenarios. However, current vision-based sensors are still not sufficiently reliable to detect imminent collisions in many conditions. Hence, a new type of compact and energy efficient vision sensor is required for collision detection in future robots and autonomous vehicles.

After millions of years of evolution, many animals possess a critical ability to escape from suddenly appearing or rapidly approaching predators or threats, due to the efficiency and robustness of their visual systems that are capable of perceiving looming objects (Gabbiani et al., 1999; Rind and Simmons, 1999; Borst and Euler, 2011; Borst and Helmstaedter, 2015; Tomsic, 2016). Compared with vertebrates, invertebrates such as arthropods have a relatively small number of visual neurons, but they can still navigate flexibly in chaotic and dynamic visual environments. Biological research has revealed a number of specialized visual neurons for detecting motion cues including looming in the visual pathways of invertebrates, for example, in locusts (Rind et al., 2016), flies (Fotowat et al., 2009), crayfish (Glantz, 1974), and praying mantis (Yamawaki and Toh, 2009). Moreover, the stereotyped behaviors produced by invertebrates are much easier to simulate and model (Webb, 2002), as are their compact visual pathways (Rind and Bramwell, 1996; Browning et al., 2009; Fotowat et al., 2009; Yamawaki and Toh, 2009; Rind et al., 2016; Wang et al., 2018, 2019).

Modeling of the biological visual systems not only helps neurobiologists and neuroethologists to further understand the underlying mechanisms, but also provides solutions for robots and autonomous systems (Clifford and Ibbotson, 2002; Yue and Rind, 2006, 2007, 2013; Hu et al., 2016; Fu et al., 2019b; Zhao et al., 2019). From the perspective of engineering, these bio-plausible models can be produced easily on very large-scale integration (VLSI) chips for high volume production (Sarkar et al., 2012; Milde et al., 2017).

Amongst the visual motion detectors in invertebrates, the lobula giant movement detectors (LGMDs, Gray et al., 2010; Rind et al., 2016), the lobula plate tangential cells (LPTCs, Borst and Euler, 2011; Fu et al., 2019a), the small target motion detector (STMD, Nordström et al., 2006; Wiederman et al., 2008; Nordström, 2012) have been the most extensively studied and modeled neurons during the last few decades. Specifically for looming sensitive neurons, the lobula plate/lobula columnar type II (LPLC2) neurons in Drosophila demonstrates ultra-selective response to expanding objects only from center of visual field (Klapoetke et al., 2017). A pair of motion-sensitive detectors, LGMD1 and LGMD2, found in locust have been shown to be sensitive to looming stimuli, each with specific selectivity (Rind and Bramwell, 1996; Rind et al., 2016). Although the locust have at least two kinds of LGMDs, there is no convincing behavioral evidence that the avoidance response to looming stimulus is highly directionally tuned (Chan and Gabbiani, 2013). Therefore, these motion-sensitive neuron models can not localize the spatial looming.

A species of crab named *Neohelice granulata* has shown a remarkable capability for detecting spatial position of predators impinging on its visual receptive field. The corresponding motion-sensitive neurons have also been identified in such crabs during the last two decades (de Astrada and Tomsic, 2002; Sztarker and Tomsic, 2004; Sztarker et al., 2005; Medan et al., 2007, 2015; Oliva and Tomsic, 2012, 2014). In crabs, the avoidance responses to looming stimuli are far from being a ballistic-like stereotyped behavior but reflect a finely tuned escape system. For example, the direction of escape is continually regulated according to changes in the spatial position of the visual stimulus (Oliva and Tomsic, 2014). This finely tuned directional escape system cannot be realized by a single motion-sensitive neuron, but is accomplished by an assembly of MLG1 neurons. In fact, a directional change in the stimulus of less than 1 deg is enough to make the running crab adjust its direction of movement (Medan et al., 2015), which means the crabs accurately identify the spatial location of the looming stimulus. Under the guidance of such a compact visual system, crabs exhibit surprising accuracy in detecting the spatial location of a looming stimulus, and this makes it an ideal model on which to fashion future robotic vision systems.

The current research on MLG1s focuses on physiological investigations (de Astrada and Tomsic, 2002; Sztarker and Tomsic, 2004; Sztarker et al., 2005; Medan et al., 2007, 2015), and encoding the neural firing response based on the approaching stimulus angular velocity (Oliva and Tomsic, 2014, 2016; Carbone et al., 2018). However, little has been done on quantitative modeling the MLG1s and their pre-synaptic via approaches from computer vision and image processing. Besides, there is no motion-sensitive neuron computation model for encoding spatial looming information. To fill this gap, we have developed a computational model of MLG1s and their pre-synaptic network to simulate the functionality of spatial localization in crabs with looming sensitive neurons. The computational model demonstrates the spatial localization capability analogous to the MLG1s in crabs and can be used by neurobiologists for testing hypotheses. It can also be integrated directly into intelligent robots for detecting spatial location and direction of approaching objects.

The paper is structured as follows. In section 2, we describe the characteristics of the MLG1 that plays a central role in the crab visual system, and outline some of the current research on bio-inspired visual motion detection models and their applications. In section 3, we propose a novel MLG1s-based bio-inspired neural network. In section 4, systematic experiments are described that prove the validity and robustness of the proposed neural network for looming spatial localization. Section 5 comprises further discussion. Section 6 concludes the paper.

## 2. Related Research

Within this section, we briefly review related research in the areas of (1) motion-sensitive giant neurons in crabs, (2) bio-inspired visual motion perception models and their applications.

### 2.1. Motion-Sensitive Giant Neurons of Crabs

According to their morphology and physiology (Medan et al., 2007; Tomsic et al., 2017), four different classes of lobula giant neurons had been described. These are monostratified lobula giant 1 and 2 neurons, and bistratified lobula giant 1 and 2 neurons (MLG1, MLG2, BLG1, and BLG2, respectively).

These are thought to be central elements involved in motion detection. Similar to LGMD1 in locust, LGs respond to object motion rather than to optic flow (Medan et al., 2007).

**Figure 1:**
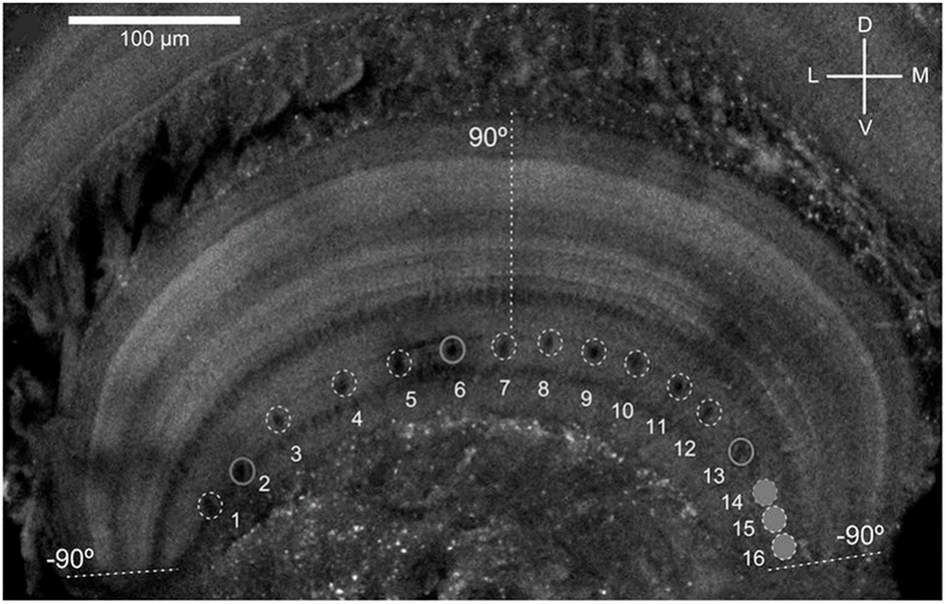
Distribution of the main dendritic profiles of the 16 MLG1s across the transversal lobula axis (see Medan et al., 2015 for details). Dashed lines indicate region of the lobula where the lateral visual pole of the eye (90°) and the medial visual pole (−90°). D, Dorsal; V, ventral; L, lateral; M, medial.

**Figure 2:**
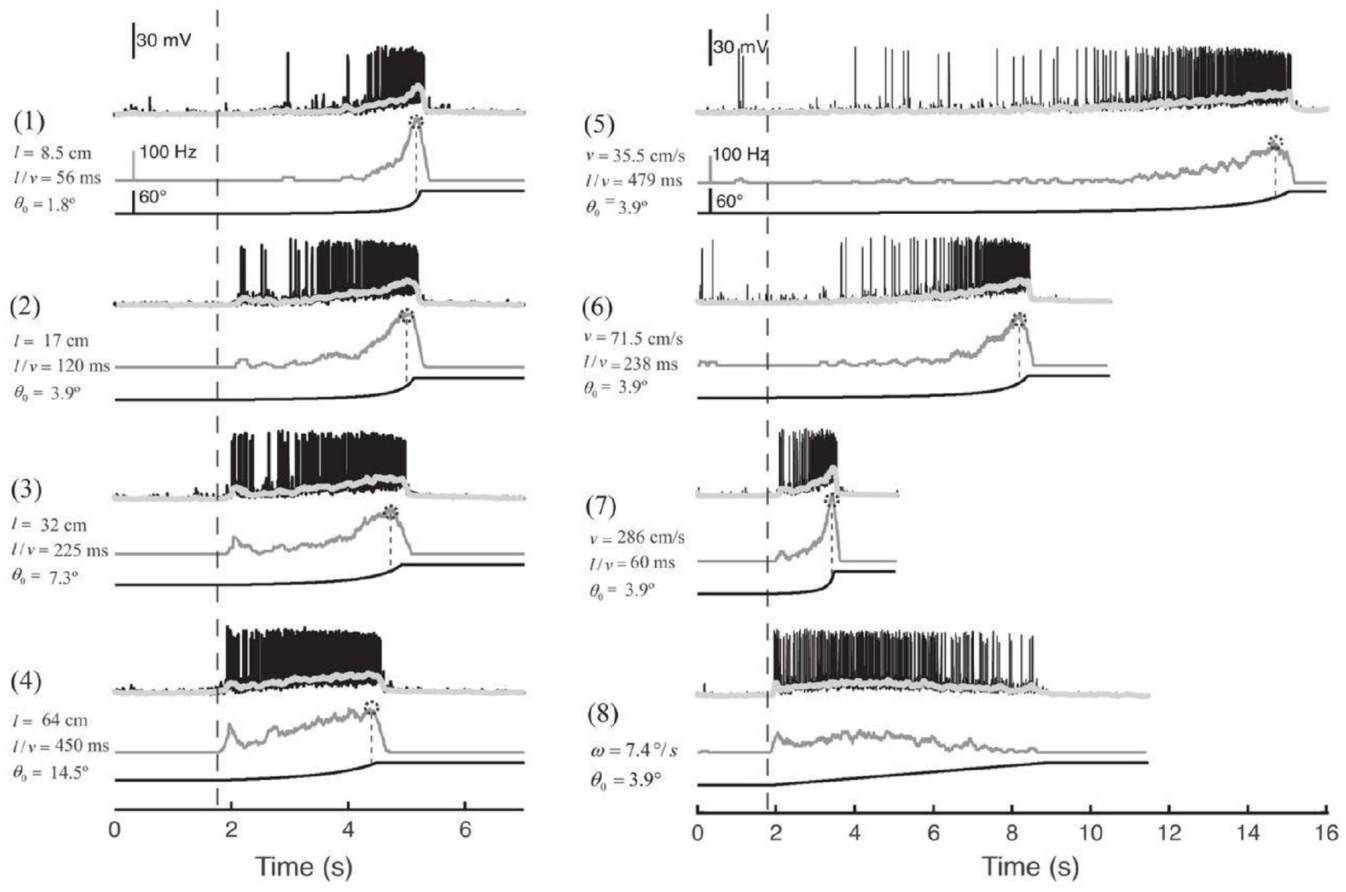
Response of an MLG1 neuron to different looming stimuli. Looming stimuli parameters (stimuli 1–7): *l* is the half-size of the object. *v* is the approach speed. θ_0_ is the initial angular size of the object in degrees. *l*/*v* is used to describe the dynamics in terms of time to collision. Black traces are real intracellular recordings from MLG1 neuron. Light gray traces are average membrane potential. Dark gray traces below correspond to the firing frequency. Dotted circles on the top of the lines are the peak of responses. The lines at the bottom are the stimuli image expansion. Dashed vertical lines signal the beginning of the stimulus expansion. Stimulus 8: black square expands at a constant angular velocity ω = 7.4*deg*/*s*, adapted from Oliva and Tomsic (2014).

As shown in Figure 1, the MLG1 neurons form an ensemble of 16 elements which are distributed on the lateromedial lobula axis where they map the 360-deg azimuthal positions of visual space (Tomsic et al., 2017). Nevertheless, with a mean receptive field of 118.4±38.9 deg, MLG1 neurons have considerable overlap with their neighboring elements (Medan et al., 2015). Morphological and physiological measurements show that more MLG1 neurons focus on the lateral area. In other words, crabs have the maximum optical resolution laterally (De Astrada et al., 2012). Because of the distributions of receiving domains, MLG1s are suited perfectly for encoding the positions of objects, which is necessary for escaping from predators directly away. In addition, MLG1s have been proved sensitive to the size and speed of the looming stimulus (Oliva and Tomsic, 2014). Figure 2 shows the responses of an MLG1 neuron to looming stimuli with differing dynamics.

Contrasting with the receptive field of MLG1 neurons, MLG2 neurons have wide and homogenous receptive fields that cover the entire visual receptive field (Oliva and Tomsic, 2014). There is apparently only one MLG2 neuron in the lobula and it has been found to encode the changes in speed of approaching stimuli. The speed change information is then conveyed downstream to the motor control system of the crab enabling them to continuously adjust their escape speed (Oliva and Tomsic, 2012). In addition, the MLG2 neuron appears to be insensitive to the spatial position of the moving object. Furthermore, no differences could be found between MLG2 responses to objects moving along the vertical or the horizontal axes (Oliva et al., 2007). Thus, during an event of approaching danger, MLG1 and MLG2 neurons are thought to continually regulate escape direction and speed, respectively, based on the observed changes of the approaching stimuli. It should be noted that MLG1 neurons have a significantly shorter latency in responding to luminance change than any other giant motion-sensitive neurons in crab (Medan et al., 2007). This could be an important factor in influencing crabs to react, timely and effectively, to directional looming stimuli corresponding to different evasive actions, which inspire us to model the MLG1s and their pre-synaptic networks initially.

### 2.2. Bio-Inspired Motion Detection Models and Applications

The monostratified lobula giant neurons (MLGs) in the crab and the LGMD1 in locust perform similarly in detecting approaching objects (Oliva et al., 2007; Oliva and Tomsic, 2014, 2016). Thus, we will mainly review the research on modeling motion-sensitive detectors inspired by LGMDs in the locust.

LGMD1 is a large visual interneuron in the optic lobe of the locust that shows a strong response to looming stimuli but little to receding stimuli (Rind and Simmons, 1992). Rind and Bramwell (1996) first built a functional neural network based on the LGMD1 input circuit. Their model mainly depends on the expanding edges of looming objects and lateral inhibition to mediate collision avoidance. Yue and Rind (2006) had further developed this neural network. They introduced a new feature to enhance the recognition of expanding edges of colliding objects. This allows the model to be used for collision detection against complex backgrounds and has since been applied successfully to vehicles to detect incoming collisions (Yue et al., 2006). To enhance the selectivity to approaching and receding objects, (Meng et al., 2009) proposed a modified LGMD1 model with additional movement information concerning direction of motion in depth. The offline tests showed improvements in efficiency and stability with little additional computational cost. Fu et al. (2018) introduced plausible ON and OFF pathways and a spike frequency adaptation mechanism to strengthen the LGMD1's collision selectivity. Very recently, (Xu et al., 2019b) propose a novel temporally irreversible visual attention model based on the implementation of central bias, LGMD, DSNs and attentional shift. With similarities to human dynamic vision, this model performs best when compared with other visual attention models. Also, it greatly reduces the computational workload (Xu et al., 2019a). Furthermore, the authors used a similar structure to devise a model to estimate human gaze positions when driving. In Drosophila, Fu and Yue (2020) proposed a computational visual pathway model to decode the translating directions against cluttered moving backgrounds. The outstanding robustness and computational simplicity are the most important features of the all these models. Hence, such collision avoidance strategies have been widely used in the application of vehicles, microrobots and UAVs (Green and Oh, 2008; Hu et al., 2016; Sabo et al., 2016; Hartbauer, 2017; Fu et al., 2018, 2019b; Zhao et al., 2018, 2019). However, how to extract spatial cues from continuously looming motions still presents serious challenges among these motion-sensitive neuron inspired models and robots.

For visual motion-sensitive neurons ensemble in crabs, (Oliva and Tomsic, 2014, 2016) proposed biophysical computational models to fit the biological responses of MLG1 and MLG2 neurons by encoding information on the stimuli and angular velocities, respectively. The results show that the computational value is in good agreement with the actual value. Stouraitis et al. (2017) demonstrated a crab robot designed to mimic the escape behavior of fiddler crabs. They created a biological compound eye model to simulate the crab ommatidium, and a color filter to extract visual cues. The effectiveness of the evasion response was then verified through different visual cues in tests. However, the capacity of MLG1 in the detection of looming objects was not mentioned in their study. To the best of our knowledge, there is no previously published computational MLG1s model to describe the looming spatial localization capabilities in crabs. In this paper, we propose a visual neural network model of the MLG1 neurons for the first time, with a focus on directional responses and looming. Compared to the introduced bio-inspired methods, this neural network model addresses the deficiency of single neuron computation in extracting local, spatial looming information, that not only fulfills the revealed characteristics of the crab's motion sensitive neural systems, but also provides an alternative effective solution to real-world collision detection-and-avoidance problems.

## 3. Framework of the Proposed MLG1s-Based Model

Although MLG1 neurons demonstrated looming spatial localization capability, the underlying mechanisms and circuits remain unclear (Oliva and Tomsic, 2014). Similar to visual nervous systems in invertebrates, crabs have retina layer, lamina layer, medulla layer, and lobula complex to process visual signals. As mentioned above, in locust, the looming sensitive neuron LGMD and its computational models have been studied extensively. In this study, we take the inspiration of LGMD neural circuits to form part of the MLG1 neuron models. Nevertheless, our proposed model characterize a winner-take-all mechanism as the post-synaptic to judge the spatial location of the approaching object and introduced a robust and bio-plausible spike frequency adaptation mechanism to enhance the selectivity to looming and receding stimuli.

### 3.1. Characteristics of Eyes and MLG1 Neurons

The crab *Neohelice granulata* has a sophisticated visual system consisting of two compound eyes. Each compound eye contains 9,000 ommatidia spherically distributed around the tip of each eyestalk to collect visual information from 360 deg in the azimuthal plane (Tomsic et al., 2017). The visual signals are transferred from the retina to the lamina, the medulla and the lobula complex, which includes the lobula and lobula plate. 16 motion-sensitive neurons (MLG1s) are almost evenly distributed in the lobula, as shown in Figure 1. Each of the MLG1 neurons have an average receptive field of 118.4±38.9 deg which means each MLG1 has considerable overlap with its neighbors (Medan et al., 2015). Studies have shown that MLG1 neurons have two characteristics: (1) as an ensemble, the MLG1s allows continual looming spatial localization, and (2) the MLG1 neuron has a higher degree of sensitivity to larger or faster approaching objects, as shown in Figure 2. In this paper, we propose a looming spatial localization detector to model these two features.

### 3.2. The Proposed Looming Spatial Localization Detector

**Figure 3:**
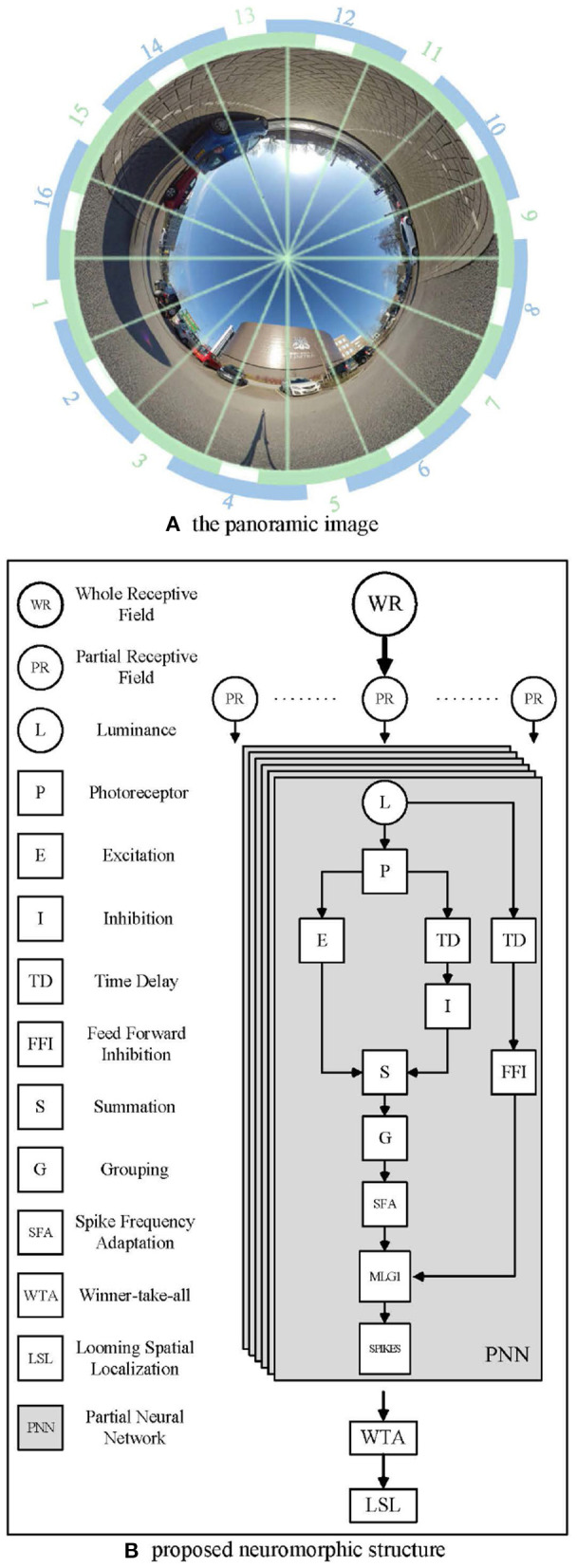
Example images from the panoramic camera. **(A)** The panoramic image divided in 16 equal segments. The proposed method involves dividing the field of view into 16 equal segments. Each unit consists of a 37.5 deg receptive field, 15 deg of which are overlapped with each neighbor. Blue and green arcs illustrate the way neighboring receptive fields overlap. **(B)** The proposed neuromorphic structure based on MLG1s for looming spatial localization. In total, sixteen identical PNNs make up the proposed MLG1s model.

Since the crab has a monocular 360 deg receptive field, we used a panoramic camera to capture images, as shown in Figure 3A. The panoramic image calibration experiment could be find in the Supplementary Material. The center of the captured image is the sky above the camera. The objects approaching from the distant horizon will be projected initially onto the panoramic image at an inner circle and move toward an outer circle. Different to natural MLG1 neurons, although our neural network models the characteristics, it divides the panoramic images into 16 equal segments. Each segment has a total field of view of 37.5 deg. Of this, 15 deg of perceptual field is shared with each neighbor, whilst the central 7.5 deg is perceived by the segment alone. Figure 3A shows the proposed method to divide up the image. Each green and blue arc represents one MLG1 neuron to encode the specific looming spatial location.

Figure 3B illustrates the proposed neuromorphic structure. WR stands for the whole receptive field, since the crab has a monocular 360 deg receptive field, we use a panoramic image representation. Partial receptive field (PR) is the view field covered by each MLG1 neuron. Each MLG1 neuron has the same neural network structure, named partial neural network (PNN) in our proposed neuromorphic structure. Generally speaking, each PNN includes: (1) a photoreceptor layer to retrieve continuous motion information, (2) inhibition and excitation layers to fulfill a biological lateral inhibition function, (3) a summation layer to combine both an inhibition cell and excitation cell, (4) a grouping layer to suppress noise, (5) an MLG1 cell to generate spikes, (6) an SFA mechanism to enhance the excitation signal to achieve sensitivity and selectivity, (7) a feed-forward inhibition cell to control the activation of each MLG1, and (8) a looming spatial localization mechanism to integrate the 16 MLG1s' outputs and judge the spatial location of an approaching object.

We choose one partial neural network of our model as an example to present its mathematical structure and principle in the following sections.

### 3.3. Photoreceptor Layer

In the proposed MLG1 motion-sensitive neuron model, the first layer consists of the photoreceptor layer arranged in a 2D-Matrix. Each pixel in successive images capture changes in gray-scaled brightness. The output *P*(*x, y, t*) is described by Equation (1),


(1) $$\begin{array}{l} P\left(x,y,t\right)=\overset{N_{p}}{\underset{i}{\sum }}a_{i}\cdot P\left(x,y,t-i\right)+L\left(x,y,t\right)-L\left(x,y,t-1\right) \end{array}$$

where *P*(*x, y, t*) is the current luminance change in pixel (*x, y*) at *t* moment. In addition, the luminance change can last for a limited number of frames *N*_*p*_. The persistence coefficient *a*_*i*_ ∈ (0, 1), and


(2) $$\begin{array}{l} a_{i}={\left(1+e^{\mu i}\right)}^{-1} \end{array}$$

where μ ∈ (−∞, +∞), *i* indicates the previous frame (*t*−*i*) counted from the current frame *t*. Equation (2) is used to simulate the attenuating effect of the first few frames on the brightness of the current frame.

### 3.4. Inhibition and Excitation Layer

The inhibition and excitation (IE) layer is made up of an inhibition cell and excitation cell. The mathematical value of the excitation cell is transferred directly from the photoreceptor layer.


(3) $$\begin{array}{l} E\left(x,y,t\right)=P\left(x,y,t\right) \end{array}$$

The lateral inhibition cell has one frame delay to its retinotopic counterpart's neighboring cells.


(4) $$\begin{array}{l} I\left(x,y,t\right)=P\left(x,y,t-1\right)\cdot W_{i} \end{array}$$

and the weightings of the convolution kernel *W*_*i*_ fit the following matrix,


(5) $$\begin{array}{l} W_{i}=\left[\begin{array}{ccc} 1/8 & 1/4 & 1/8\\ 1/4 & 0 & 1/4\\ 1/8 & 1/4 & 1/8 \end{array}\right] \end{array}$$

### 3.5. Summation Layer

The output of the summation layer is the sum of the inhibition and excitation layers, i.e.,


(6) $$\begin{array}{l} S\left(x,y,t\right)=E\left(x,y,t\right)+I\left(x,y,t\right)\cdot W_{I} \end{array}$$

where *W*_*I*_ is the weight of inhibition.

### 3.6. Grouping Layer

In this proposed MLG1 based neural network, in order to extract collision targets against complex backgrounds, the extended edge represented by cluster excitation needs to be enhanced. Therefore, a passing coefficient, *Ce*(*x, y, t*) has been applied to the grouping layer to multiply the excitations. It should be noticed that the grouping layer is a mathematical filter with no physiological basis.

The coefficient is determined by the cell's surrounding neighbors:


(7) $$\begin{array}{l} Ce\left(x,y,t\right)=\overset{r}{\underset{i=-r}{\sum }}\overset{r}{\underset{j=-r}{\sum }}S\left(x+i,y+j,t\right)\cdot W_{e}\left(i,j\right) \end{array}$$

where *W*_*e*_ is an equal-weighted kernel. While in this model, we set *r* = 1, which means *W*_*e*_ is set to:


(8) $$\begin{array}{l} W_{e}=\frac{1}{9}\left[\begin{array}{ccc} 1 & 1 & 1\\ 1 & 1 & 1\\ 1 & 1 & 1 \end{array}\right] \end{array}$$

In the meantime, using the equal-weighted matrix *W*_*e*_ can also restrain the isolated noise in G layer to some extent.

Therefore, the excitation in the location (*x, y*), $\tilde{G}\left(x,y,t\right)$ can be computationally presented as:


(9) $$\begin{array}{l} \tilde{G}\left(x,y,t\right)=S\left(x,y,t\right)\cdot Ce\left(x,y,t\right)\cdot \omega^{-1} \end{array}$$

where ω is a scale and calculated at every frame as:


(10) $$\begin{array}{l} \omega =△c+max\left(abs\left[Ce\left(x,y,t\right)\right]\right)C_{w}^{-1} \end{array}$$

In the above Equation (10), △*c* is a small real number, *C*_*w*_ is a constant positive coefficient. In consequence, ω ∈ (0, +∞) and $\tilde{G}\left(x,y,t\right)$ is positively correlated with *C*_*w*_.

In the grouping layer, we also set a threshold to suppress the decayed excitations.


(11) $$G(x,y,t)=\{\begin{array}{cc} G(x,y,t) & \;if\;\tilde{G}(x,y,t)\geq T_{g}\\ 0 & \;otherwise \end{array}$$

where *G*(*x, y, t*) is the value of the excited pixels that have passed the threshold *T*_*g*_ at time *t*.

### 3.7. MLG1 Cell

The membrane potential of the MLG1 cell, *m*(*t*), is the sum of each pixel output from the grouping layer with a rectifying operation, which can be represented as


(12) $$\begin{array}{l} m\left(t\right)=\overset{row}{\underset{x=1}{\sum }}\overset{col}{\underset{y=1}{\sum }}abs\left(\tilde{G}\left(x,y,t\right)\right) \end{array}$$

The membrane potential of the MLG1, *m*(*t*) is then mapped to the sigmoid function to simulate the activation of the artificial neurons. The equation is computed as follows:


(13) $$\begin{array}{l} M\left(t\right)={\left(1+e^{-c\left(t\right)\cdot m\left(t\right)\cdot n_{cell}^{-1}}\right)}^{-1} \end{array}$$

*c*(*t*) is an adaptive coefficient which refers to the derivative of *m*(*t*), and its value range is (0, +∞). Since *m*(*t*) and *c*(*t*) are each greater than zero, the normalized spikes, *M*(*t*) ∈ (0.5, 1].

### 3.8. Spike Frequency Adaptation Mechanism

As presented in Equation (14), the biophysical spike frequency adaptation (SFA) mechanism has been introduced to enhance the model's sensitivity to looming and receding stimuli. Previous research revealed an intrinsic neural property of such looming-sensitive neurons, the SFA mechanism, which enables the neurons to discriminate approaching versus receding and translating (Fabrizio and Krapp, 2007; Peron and Gabbiani, 2009a,b). In such biophysical theory, the SFA could be illustrated as, when the motion-sensitive neuron challenged by an approaching stimulus, the continuously expanding edges activate an increasing number of excitatory synaptic inputs (i.e., photoreceptors in our proposed model) (Fabrizio and Krapp, 2007). This overcome the SFA in neurons. Our proposed computational function serves to enhance the signals from looming stimuli with a positive derivative coefficient, or inhibits signals from receding stimuli rapidly. *C*(*t*) can be mathematically defined as:


(14) $$c(t)=\{\begin{array}{cc} \Delta c & if\;c(t)\leq 0\;\\ c(t-1)+c_{i1} & if\;m(t{)}^{\prime }>0\;\&\;m(t{)}^{\prime \prime }\geq 0\\ c(t-1)+c_{i2} & if\;m(t{)}^{\prime }>0\;\&\;m(t{)}^{\prime \prime }<0\\ c(t-1)-c_{a} & if\;m(t{)}^{\prime }\leq 0 \end{array}$$

Where △*c* is a small real number and *c*(*t*) is affected by its previous moment *c*(*t*−1), incentive coefficient *c*_*i*1_, *c*_*i*2_ and attenuation coefficient *c*_*a*_.

It should be noted that the digital signal has no continuous derivative. We calculate the gradient by comparing the signals of consecutive discrete frames. So, *m*(*t*)' and *m*(*t*)” can be defined in Equation (15) and (16), respectively,


(15) $$\begin{array}{l} {m\left(t\right)}^{\prime }=\frac{\text{d}m\left(t\right)}{\text{d}t}=\frac{m\left(t\right)-m\left(t-1\right)}{\tau } \end{array}$$

(16) $$\begin{array}{l} {m\left(t\right)}^{\prime \prime }=\frac{{\text{d}}^{2}m\left(t\right)}{\text{d}t^{2}}=\frac{m\left(t\right)+m\left(t-2\right)-2\cdot m\left(t\right)}{\tau^{2}} \end{array}$$

where τ is the time constant which depends on the camera frame rate. Compared with the SFA mechanism proposed by Fu et al. (2018), our method responds to motion changes more quickly.

### 3.9. The Feed Forward Inhibition Mechanism

The feed forward inhibition (FFI) mechanism is introduced to adapt the threshold in response to a sudden change in the whole visual receptive field. The feed forward inhibition and lateral inhibition work together to handle such whole scene movements:


(17) $$\begin{array}{l} F\left(t\right)=\overset{N_{a}}{\underset{j}{\sum }}a_{j}F\left(t-j\right)+\overset{n_{r}}{\underset{x=1}{\sum }}\overset{n_{c}}{\underset{y=1}{\sum }}abs\left(P\left(x,y,t-1\right)\right)n_{cell}^{-1} \end{array}$$

where *a*_*j*_ is the persistence coefficient for FFI and its range of value is (0, 1). *N*_*a*_ defines how many time steps the persistence can last.

Once *F*(*t*) exceeds its threshold *T*_*FFI*_(*t*), spikes in the MLG1 model are inhibited immediately. It is defined by formula (18)


(18) $$\begin{array}{l} T_{FFI}\left(t\right)=T_{F0}+a_{ffi}T_{FFI}\left(t-1\right) \end{array}$$

where *T*_*F*0_ is the initial value of *T*_*FFI*_. The adaptable threshold is determined by the previous *T*_*FFI*_(*t*−1). *a*_*ffi*_ is a coefficient.

### 3.10. Spike Unit

The crab's escape behavior is initiated once MLG1 neurons have been activated, which means the looming spatial location has been already confirmed. However, neuron spikes may be suppressed by the feed forward inhibition (FFI) mechanism when the background changes dramatically. So, after MLG1 and FFI mechanisms, the final spike will be generated if *M*_*t*_ exceed its threshold *T*_*s*_ and *F*(*t*) doesn't exceed its threshold *T*_*FFI*_(*t*), respectively, i.e.,


(19) $$S^{spike}(t)=\{\begin{array}{ll} 1 & if\;M(t)\geq T_{s}\;\&\;F(t)<T_{FFI}(t)\\ 0 & otherwise \end{array}$$

where 1 represents a spike, 0 means no spike. *F*(*t*) is a global scene change judgment mechanism, and its *T*_*FFI*_(*t*) threshold value is also adaptive.

A final collision alarm signal *C*(*t*) will be produced if the number of $S_{t}^{spike}$ which are continuously excited, exceeds the set number of time steps, as defined in Equation (20),


(20) $$C_{j}(t)=\{\begin{array}{cc} 1 & \;if\sum_{i=t-N_{t}}^{t}S^{spike}(t)\geq n_{sp}\\ 0 & otherwise \end{array}$$

where *n*_*sp*_ and *N*_*t*_ denote the number of successive spikes and frames, respectively. *j* indicates the segment number of each MLG1 neuron. *n*_*sp*_ is greater than *N*_*t*_ in this model, as the exponential mapping from membrane potential to firing rate.

### 3.11. The Looming Spatial Localization Mechanism

**Figure 4:**
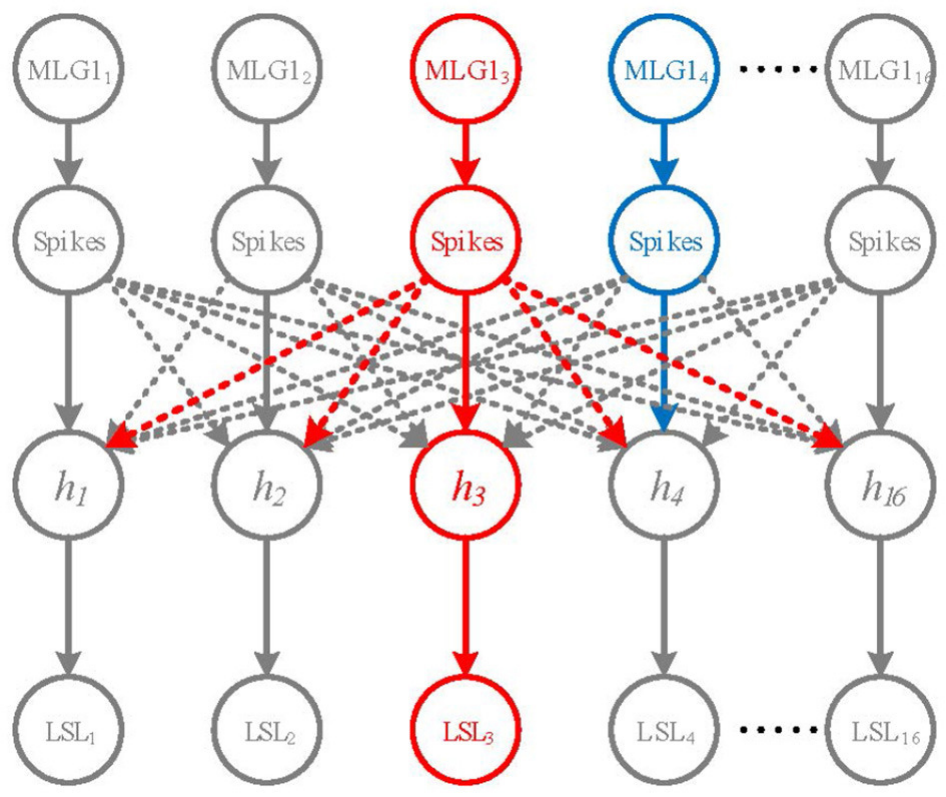
A Winner-take-all network for looming spatial localization. The solid arrow lines and dashed lines represent excitation and inhibition, respectively. The hidden layer contains 16 neurons *h*_1_ ~ *h*_16_. The red neuron *MLG*1_3_ represents the first activated neuron, the blue neuron *MLG*1_4_ is the second one. *MLG*1_3_ firstly activates *h*_3_ and inhibits the rest of hidden neurons. Thus, the output of LSL layer is *LSL*_3_.

To determine the specific spatial location of an approaching object, MLG1s may need to inhibit each other to win a competition. Although lateral inhibitions between MLG1 neurons in crabs are not confirmed, recent research suggested that there are connections between motion-sensitive neurons in the same lobula layer [e.g., MLG2 and BLG2 (Cámera et al., 2020)]. In this study, we propose a winner-take-all (WTA) network based on the early spike timing to determine the spatial location of the approaching object, as shown in Figure 4.

The WTA network consists of the inputs layer (i.e., the 16 MLG1s), a hidden layer and a looming spatial localization (LSL) layer with excitatory and inhibitory synaptic connections, as shown in Figure 4. The hidden layer receives excitation from corresponding MLG1 neurons and inhibition from other MLG1 neurons. When a spike of a MLG1 neuron (e.g., red neuron *MLG*1_3_ in Figure 4), is generated at a time *t*, the corresponding hidden layer neuron (i.e., *h*_3_ neuron) will be activated, so the output of LSL is *LSL*_3_. The other hidden layer neurons will be suppressed. Even if another MLG1 (e.g., blue neuron *MLG*1_4_) fires in the next moment, its corresponding hidden neuron will not be activated. The output of LSL could be defined as:


(21) $$\begin{array}{l} LSL_{output}=LSL_{i},C_{i}\left(t\right)=max\left({Ĉ}_{j}\left(t\right)\right) \end{array}$$

where *i* is the index number of the activated MLG1 neuron. Ĉ(*t*) could be calculated at every frames as:


(22) $$\begin{array}{l} {Ĉ}_{j}\left(t\right)=\alpha_{1}C_{j}\left(t\right)+\alpha_{2}C_{j}\left(t-1\right)+\cdots +\alpha_{n}C_{j}\left(t-n\right),C_{j}\left(t\right)\neq 0 \end{array}$$

where 0 < α_1_ < α_2_ < ⋯ < α_*n*_, α_1_ + α_2_ + ⋯+α_*n*_ = 1. *j* is the number of MLG1 neuron (*j* ∈ [1, 16]) and *n* represents frames in a time window. If the approaching stimulus activates two neighboring MLG1s at the same time, their Ĉ_*j*_(*t*) should be equal. Thus, the approaching location would be treated as in the middle of the two neighboring MLG1s.

In general, our proposed LSL mechanism indicates the spatial-temporally encoded local approaching motions at a specific moment. The post-synaptic neural network for global motions perception is outside the scope of this paper and will be investigated in our future work.

## 4. Experiments and Results

This section presents five experiments to test the feasibility and robustness of the proposed looming spatial localization neural network.

The main objectives are as follows: (1) To examine the effectiveness of the proposed looming spatial localization detector in collision detection. (2) To test the sensitivity and selectivity of the proposed model while handling looming stimuli with different dynamics. (3) To test sensitivity to contrast and the inhibition to a receding stimulus. (4) A self-rotating video is used in this subsection to examine the selectivity of the FFI mechanism. (5) To verify the effectiveness and robustness of the MLG1s model in a realistic urban scene.

### 4.1. Parameters Setting

Parameters of the proposed model have been determined according to preliminary experiments carried out for the implementation and optimization of functions. All parameter settings and descriptions are shown in Table 1.

**Table 1:** The predefined parameters and descriptions of proposed model.

**Parameter**	**Description**	**Value**
*N*_*p*_	Luminance persistence in frames Eq.1	1
*W*_*I*_	Inhibition weight Eq.6	0.3
*C*_*w*_	Constant positive coefficient Eq.10	4
△*c*	Small real number Eq.10&14	0.01
*T*_*g*_	Threshold Eq.11	30
*c*_*i*1_	Incentive coefficient Eq.14	0.5
*c*_*i*2_	Incentive coefficient Eq.14	0.3
*c*_*a*_	Attenuation coefficient Eq.14	0.3
τ	Time constant Eq.15&Eq.16	0.04
*T*_*s*_	Spike threshold 19	0.7
*n*_*sp*_	Number of spikes Eq.20	4-5
*N*_*a*_	Luminance persistence Eq.17	1
*T*_*F*0_	Initial value of *T*_*ffi*_ Eq.18	15
*a*_*ffi*_	Coefficient Eq.18	0.02

All the experimental videos are real-world looming stimuli against a cluttered background which contains shadows, reflections, different gray-scaled objects, etc. The videos are recorded by the panoramic camera, Insta360 ONE X (see Ins, 2021), to imitate the crab's eyes. The camera operates at 30 fps and the video resolution is 720*720 in all the experiments. It should be noted that the video resolution doesn't affect the results of experiments a lot. Higher resolution means more computation, in other words, it takes longer to produce results.

All experiments are conducted on a Windows 10 platform with a PC [CPU: Intel (R) Core (TM) i7-4770 CPU @ 3.40 GHz, RAM: 16GB]. Data analysis and visualizations have been implemented in Matlab R2019b (The MathWorks, Inc. Natick, USA).

### 4.2. Detection of Looming Spatial Location

**Figure 5:**
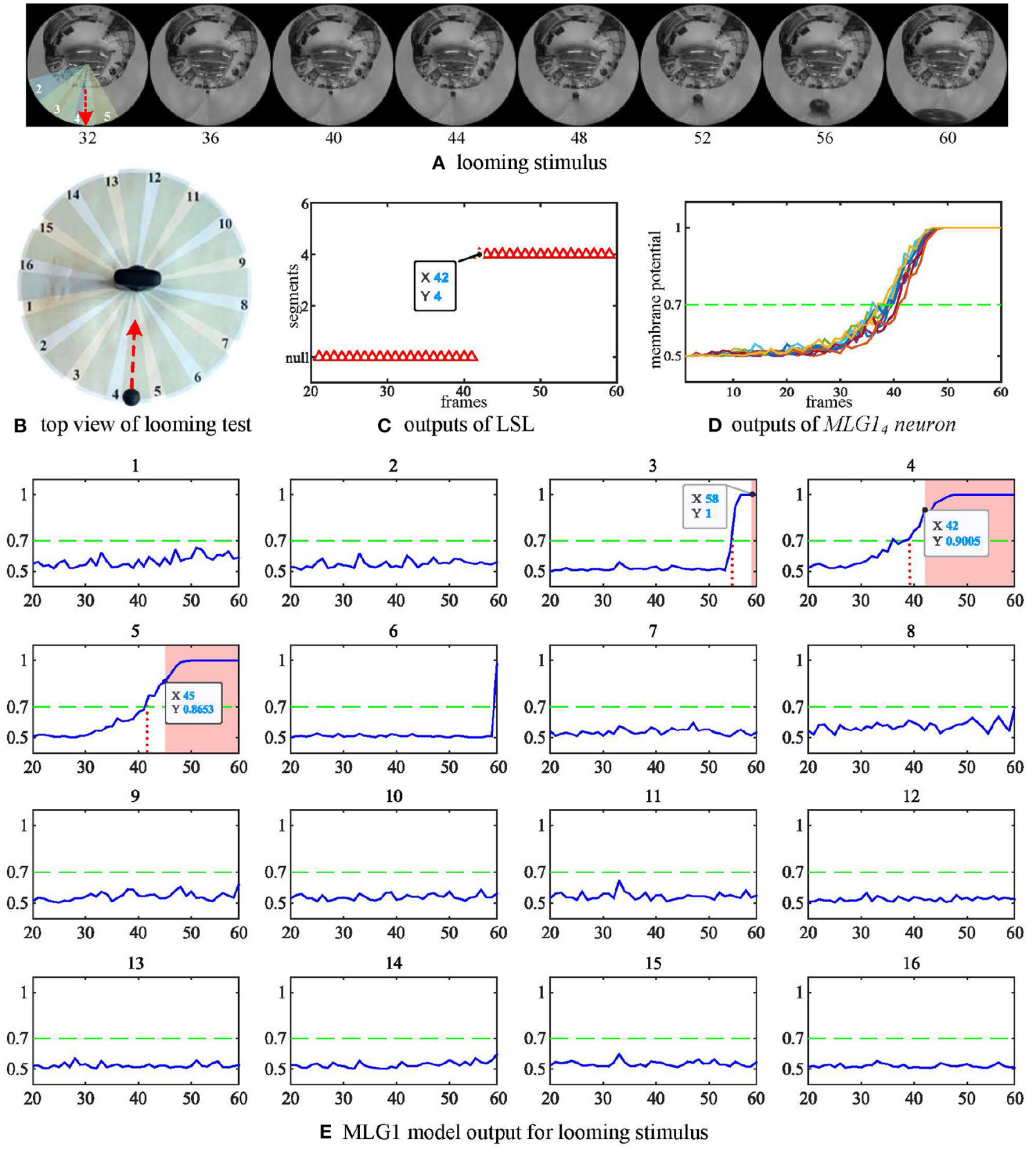
Response of the proposed model to a looming stimulus. **(A)** Looming stimulus. The collision event occurred at the 60th frame. The red dashed line is the looming trajectory. **(B)** Top view of looming test. The red dashed line is the looming trajectory as same as in **(A)**. **(C)** The first outputs of LSL unit in this test. The LSL has no output until 42nd frame (i.e., *MLG*1_4_ firstly generates spike at this frame). **(D)** Outputs from *MLG*1_4_ neuron when challenged by repeated identical looming tests. The looming stimuli start from same position at similar speeds. **(E)** The response of the 16 MLG1 neurons from our proposed model. The blue lines are membrane potential of MLG1 neurons. Red area is collision warning. The number of each subgraph corresponds to the segment number within the proposed structure in **(B)** and Figure 3A. The Y-axis denotes a normalized membrane potential and the X-axis denotes the number of frames. The red vertical dashed line indicates the time at which the response output exceeds the threshold. The red area is the time window in which each MLG1 neuron generates spikes.

For this, which is the most important characteristic, we first examine if the proposed model can recognize the spatial location of the approaching stimulus. In this experiment, we fixed the panoramic camera on the desktop and used a black ball to approach the camera directly along the track and finally collide with the camera. The red dashed line is the trajectory of looming in Figure 5A. The experiment's top view has been shown in Figure 5B. As shown in Figure 5E, each subgraph represents one MLG1 which corresponds to the equivalent segment number of the structure in Figure 3A. The activities of MLG1s represent their signaling of the looming events. The results illustrate the response of our proposed MLG1s model to a looming stimulus mainly located in segments 3–5. *MLG*1_4_ is the main response neuron which exceeds the threshold at 39th frame, and the collision warning (red area in coordinate images) is generated at 42nd frame. In *MLG*1_5_ the threshold response occurs at the 42nd frame and the collision warning occurs at the 45th frame. In *MLG*1_3_ the threshold response warning occurs at the 58th frame whilst the collision occurs at the 60th frame. The other neurons produce no warnings. In other words, since *MLG*1_4_ first perceives the looming stimulus, the LSL judge the spatial location of the looming stimulus is approximately located in the receptive field corresponding to *MLG*1_4_. And the output has been shown in Figure 5C. In addition, the similar responses shown during ten repeat experiments, (Figure 5D) illustrate that the model is very robust. Our results in the aforementioned figures show that the proposed MLG1 neurons model could successfully detect the spatial location of looming stimuli.

**Figure 6:**
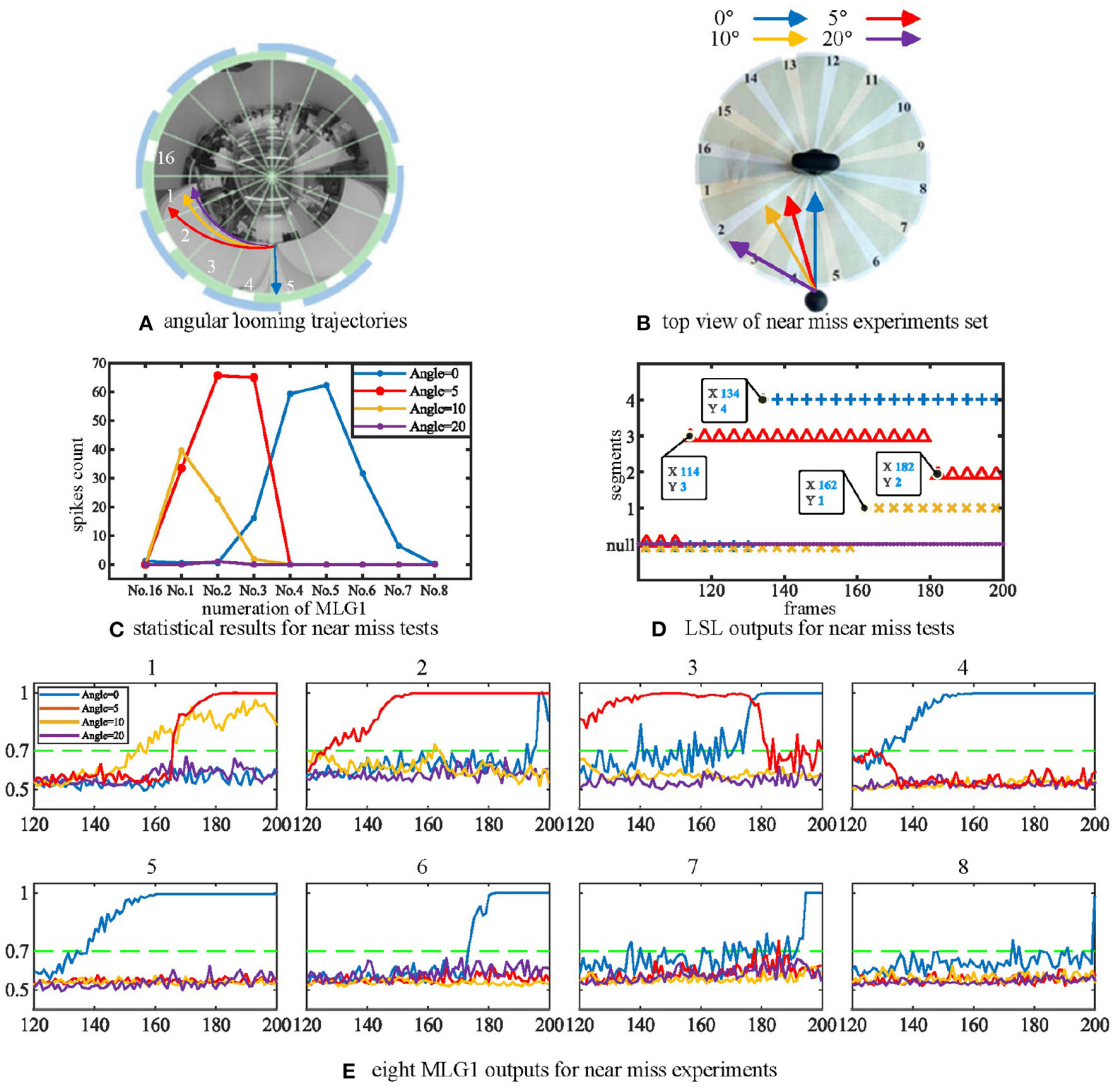
**(A)** The angular looming experiments set. The blue, red, yellow and purple lines represent angular looming trajectories with angles 0, 5, 10, and 20 deg, respectively. Different approach angles make the images of the balls travel through various segments of the receptive fields of view. **(B)** Top view of the angular looming test. **(C)** statistical results for angular looming experiments. The Y-axis denotes the spike count. The X-axis shows the numeration of MLG1 neurons, which is the same as in Figure 3A. Different looming trajectories excite corresponding MLG1 neurons. However, as the looming angles increase, neuron reactions decrease. Each experiment is repeated ten times at each angle. **(D)** the outputs of LSL in each of four angular looming tests. **(E)** eight MLG1 outputs for angular looming experiments. The X-axis is the number of frames. The Y-axis donates a normalized membrane potential. It should be noted that the closest object has a significant distortion in the panoramic image, so that it will elicit responses by adjacent neurons.

We have also investigated the performance of the model using non-collision. Figure 6A shows the image of four looming trajectories with different angles of approach. The experimental videos can be found on our Github. The statistical results in Figure 6C demonstrate that the direct looming stimulus (0 deg, blue line) creates the highest spike counts in *MLG*1_4_ and *MLG*1_5_. With an approaching angle of 5 deg (red line), the highest spike counts appear in *MLG*1_2_. As the approaching angle increases (yellow and purple lines), so does the distance between the stimulus and the camera, which results in lower spike counts. This suggests that the angular looming actions can also activate the MLG1s neural network, but the spike frequency falls off considerably when the object moves to pass with a significant deviation. Figure 6C demonstrates that the main segments where the approaching event occurred, but it can't show when the approaching event began or ended. Figure 6E shows the eight MLG1s' outputs when challenged with four kinds of angular looming. When the approaching angle is 0 deg (blue lines in the Figure 6E), the output of *MLG*1_4_ firstly exceeds the threshold (0.7) for four successive frames at the 134th frame. *MLG*1_5_ spikes at the 142nd frame. Therefore, the LSL unit confirms that the location of the approaching ball firstly comes from *LSL*_4_. As the ball continues approaching, it distorts heavily in the captured images and appears in segments 3 and 6. However, the outputs of neighboring *MLG*1_3_ and *MLG*1_6_ will not contribute to the approaching location judgement as well, even though their outputs exceed the threshold. When the looming angle is 5 deg away from the direct collision course (0 deg here), the outputs of *MLG*1_3_ firstly exceed the threshold in four successive frames. The approaching location is therefore determined by *LSL*_3_. Although the outputs of *MLG*1_2_ and *MLG*1_1_ exceed the threshold gradually later, they will not change the perceived original approaching angle already decided by *LSL*_3_. As the time goes on to 182nd frame, the ball passes through segment 3. The *LSL*_3_ then has been released, *MLG*1_1_ and *MLG*2_2_ still have been activated. Because of the WTA-based LSL mechanism, the output of LSL is *LSL*_2_. When the approaching angle is with 10 deg deviation, it only activates *MLG*1_1_ successfully in this single test as illustrated in yellow lines in Figure 6E, so the approaching location is *LSL*_1_. When the looming angle is with 20 deg deviation, no MLG1 of our proposed model responds to it. The LSL outputs for this angular looming test has shown in Figure 6D.

### 4.3. Multiple Size and Looming Velocity Tests

**Figure 7:**
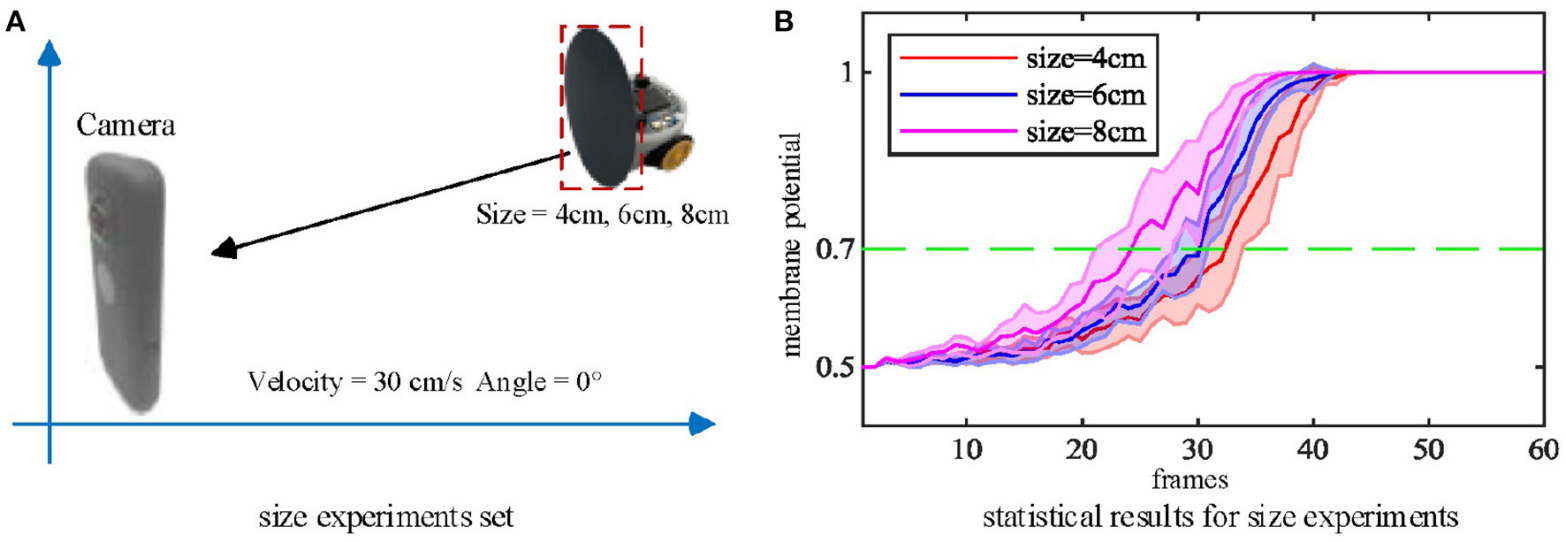
**(A)** Three objects of different sizes are used as the looming stimuli. **(B)** Statistical result (shaded error bar) shows that the larger looming objects will lead to an earlier neural response. Each experiment is repeated ten times at each objective size.

Secondly, we tested the effect of the looming object size on the model output. The microrobot Colias proposed by Hu et al. (2018) is used as a looming physical stimulus in this experiment. As shown in Figure 7, a group of comparative experiments are conducted with stimuli diameters of 4 cm, 6 cm, and 8 cm. The largest size stimulus has the earliest spike tendency. The neural response of the proposed model also matches the biological characteristics in Figure 2(1)–(4).

**Figure 8:**
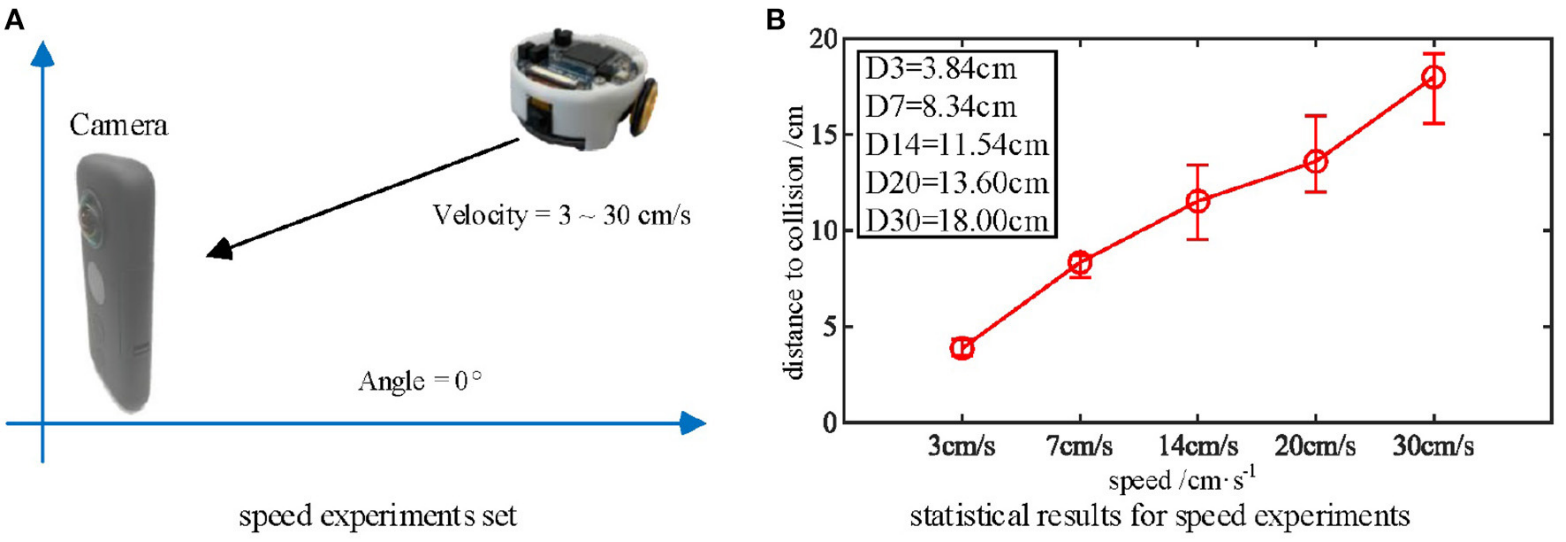
**(A)** Five approaching objects with different speeds are used as the stimuli. **(B)** Statistical results (error bars) shows that the looming stimulus with the highest velocity has the longest warning distance in these comparative experiments. Each experiment is repeated five times at each speed.

As mentioned above, the biological research confirms that MLG1 neurons are sensitive to looming speed (Oliva and Tomsic, 2014). Thus, we devised a series of experiments to show the influence of the looming speed with distance-to-collision (DTC). It should be noted that the speed of the Colias robots could not be maintained at an identical level in each single test, even though the speed rate parameter is the same. As illustrated in Figure 8, we employed five gradually increasing speeds from 3 cm/s to 30 cm/s. The statistical result shows that the DTC increases when the looming speed increases. At the looming speed of 3 cm/s, the average DTC has been issued in the ten repetitions is 3.86 cm. When the looming speed is 30 cm/s, the average distance has increased to 18.00 cm. The neural response is consistent with biological studies on the characteristics of MLG1 neurons as shown in Figure 2(5)–(7).

### 4.4. Multiple Contrast Tests and SFA Test

**Figure 9:**
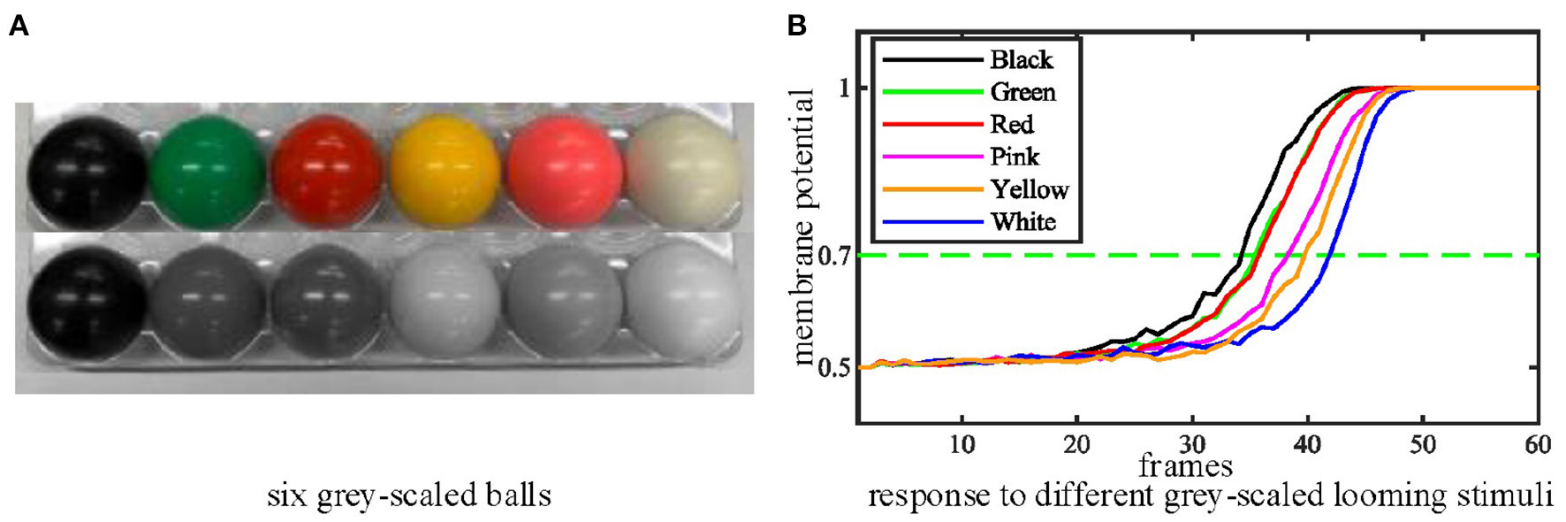
**(A)** Six grey-scaled balls. **(B)** The statistical results of contrast tests. The looming stimulus with the highest contrast responds soonest. Each experiment is repeated ten times.

Thirdly, we tested the performance of the proposed MLG1s model with stimuli exhibiting a range of contrasts to examine whether the contrast influences the MLG1s model response to collision events. We move the ball directly toward the camera at a standard velocity in these tests. Figure 9A shows six colored balls and their gray levels. The statistical results of contrast tests in Figure 9B demonstrates that the proposed MLG1s model is sensitive to the contrast between the looming stimulus and the background. As the balls approach, the MLG1s model responds earliest to the black ball which creates maximum contrast with the background. The green and red balls with similar, medium contrast levels result in approximate neural responses. The pink, yellow, and white balls, which gradually present lower contrasts, cause the responses to take progressively longer.

**Figure 10:**
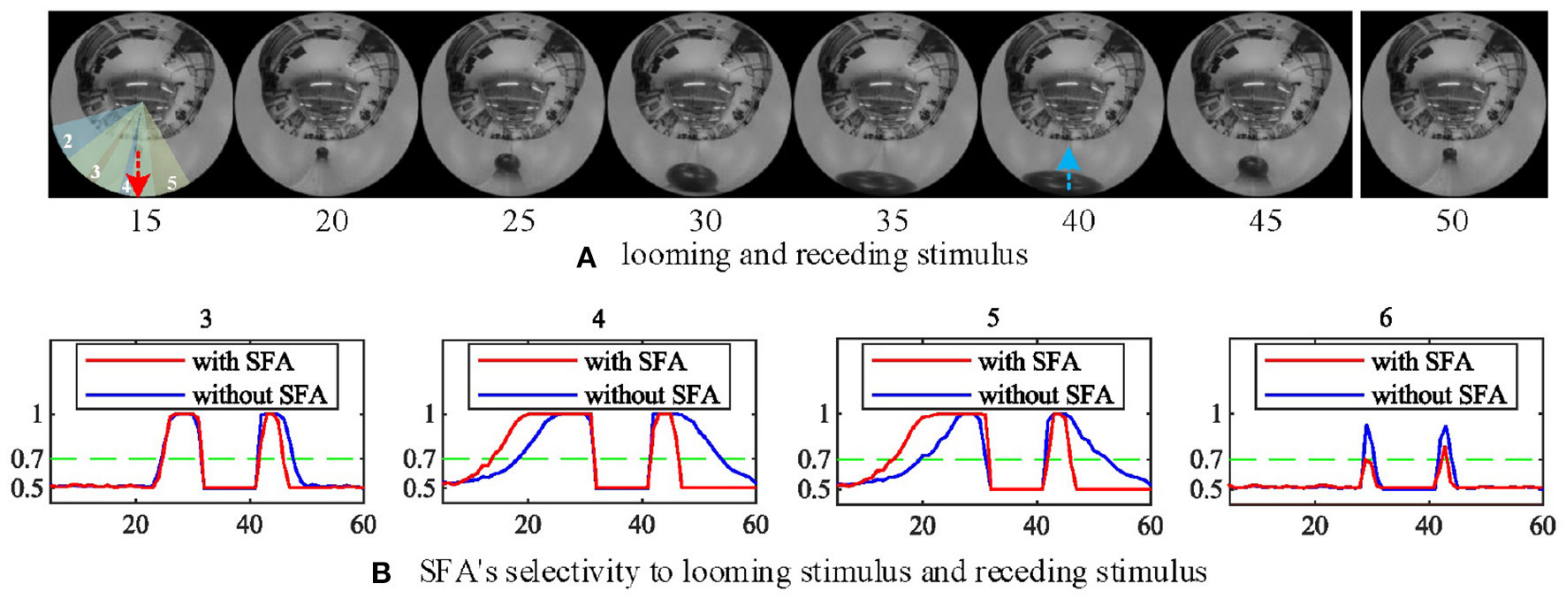
The selectivity of the SFA mechanism to looming and receding stimuli. **(A)** Experimental video containing looming and receding stimuli. The motions are mainly located in areas covered by *MLG*1_4_ and *MLG*1_5_. The red and blue dashed lines represent the approaching and leaving trajectory, respectively. **(B)** The result of *MLG*1_4_ and *MLG*1_5_ show the model with the SFA mechanism that improves the neural response generated by the looming stimuli while inhibiting the response during the recession. In *MLG*1_3_ and *MLG*1_6_, the SFA has little impact on the output because they are not the main area where the movements take place. The number of each subgraph corresponds to the segment number of the proposed structure in Figure 3A. The Y-axis denotes a normalized membrane potential and the X-axis is the number of frames.

**Figure 11:**
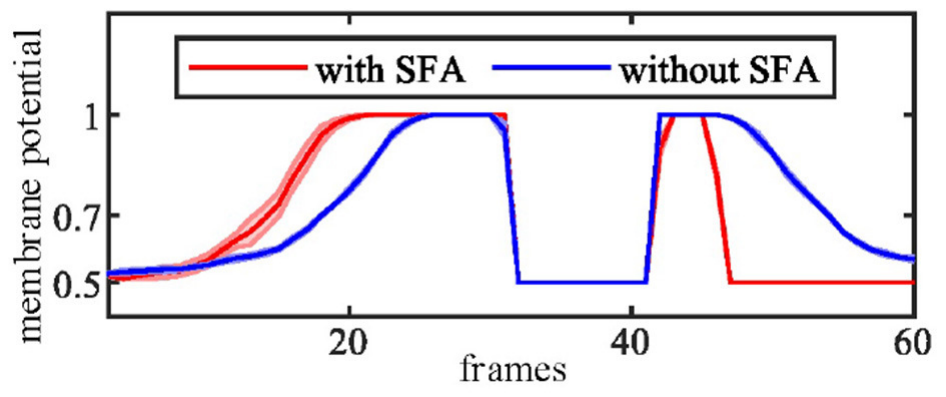
The *MLG*1_4_ response to looming and receding stimuli with the SFA mechanism. Looming occurred between 10–30 frames, receding 40–50 frames. The trajectories of the object in the stimuli are similar to those in Figure 10A. The results are overlaid from the same experiment repeated ten times.

In this group of experiments, we examined the selectivity of the SFA mechanism by using experimental videos containing both looming and receding stimuli. The movements mainly occurred in segment 3 and segment 4, so the neural response to looming is more pronounced in these two neurons. Our results in Figure 10B show that when challenged by a looming stimulus, the SFA mechanism enhanced the model's response, so the output is amplified. On the other hand, when challenged by receding stimulus, the SFA mechanism inhibited the response causing it to decay more quickly. In short, the SFA mechanism has been proven to identify and selectively modulate the response to approaching and receding stimuli. Like the repeated identical looming tests, we use a group of receding videos to examine our proposed model. Figure 11 is the results of ten times looming and receding tests. The outputs of *MLG*1_4_ from the 40th frames to around 47th frames are very close due to the similar positions of the balls and tracks used in the repeated experiments and the fast inhibitory effect of the SFA mechanism. It can also prove that the function of receding inhibition in our proposed model is very robust when handling the receding stimulus.

### 4.5. Rotational Visual Stimuli Test

In all of our previous experiments, we have the panoramic camera fixed to the table, so the camera doesn't move or wobble or rotate. If we want to embed this model in autonomous microrobots with the function of collision detection and avoidance, we must inhibit the false spikes caused by the microrobot's rotations or fast turning. So, we set up an FFI mechanism to inhibit the output at these critical moments.

**Figure 12:**
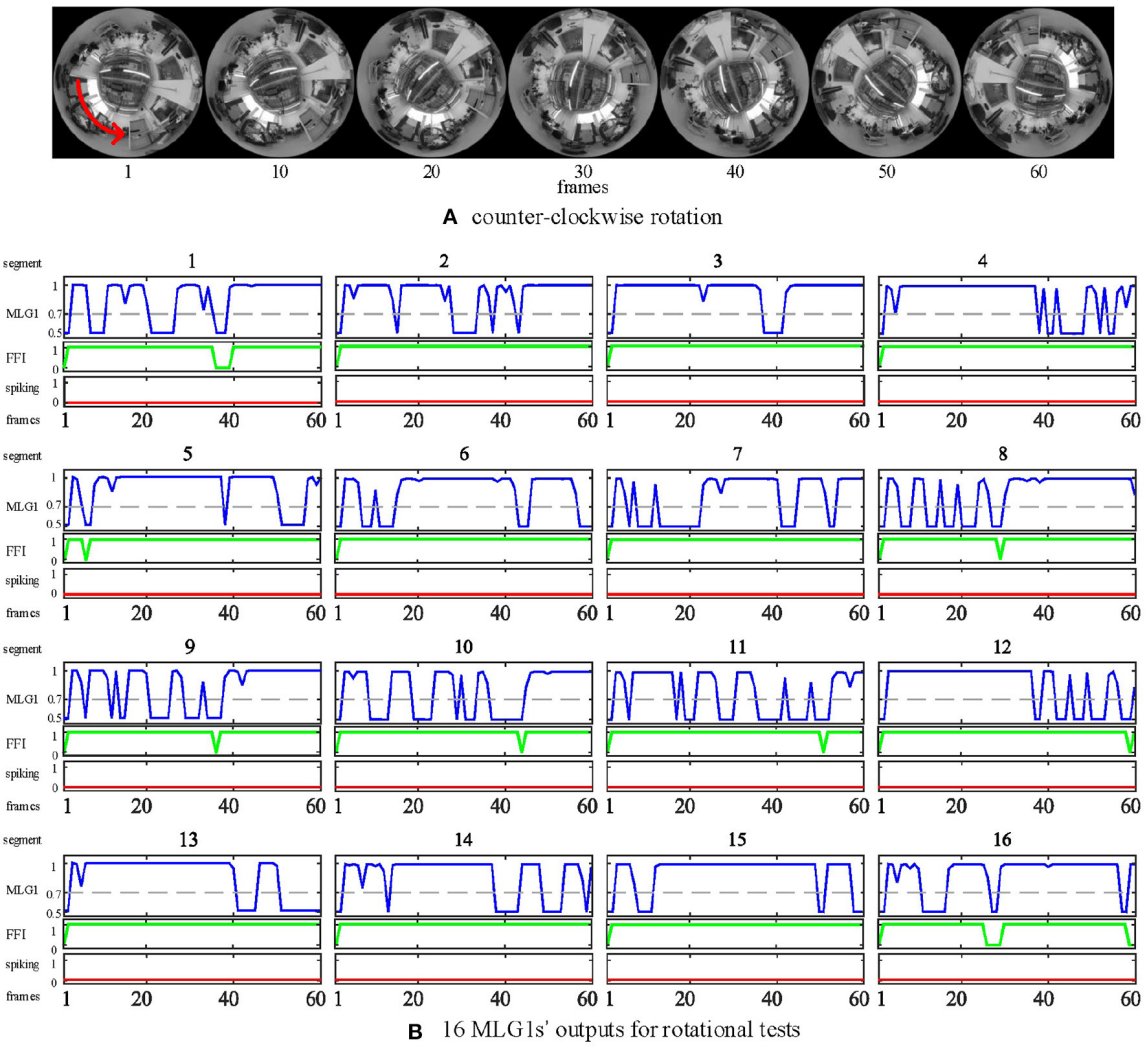
Response of the proposed model to rotational motion. **(A)** counter-clockwise rotation. The panoramic camera rotates clockwise to simulate rotational movements. The red arrow is the direction of the stimuli resulting from camera rotation. **(B)** the outputs of rotational tests from all 16 MLG1s. The blue lines are the membrane potential outputs of MLG1 neurons. The green lines are the outputs of FFI. The red lines are spiking outputs from every MLG1 neuron, representing the collision warning. The FFI function can inhibit spikes caused by rotation efficiently. The number on the top of each subgraph corresponds to the segment number within the proposed structure in Figure 3A.

In the rotational tests, we examined the selectivity of the FFI mechanism by using a video captured while the camera is rotating. In this experiment, the panoramic camera rotates clockwise at a constant angular speed of around 90 deg per second. The red arrow in Figure 12A is the direction of rotation. Our results in Figure 12B show that when the camera rotates violently, the FFI mechanisms in MLG1 neurons have been activated immediately. Thus, there are no spikes in MLG1s.

Although the FFI mechanisms perform well in the rotational test, we still consider the FFIs as a whole. For example, when 10 of the 16 FFIs are activated, the model determines that it is self-rotating and then inhibits all MLG1s from spiking.

### 4.6. Urban Scene Test

**Figure 13:**
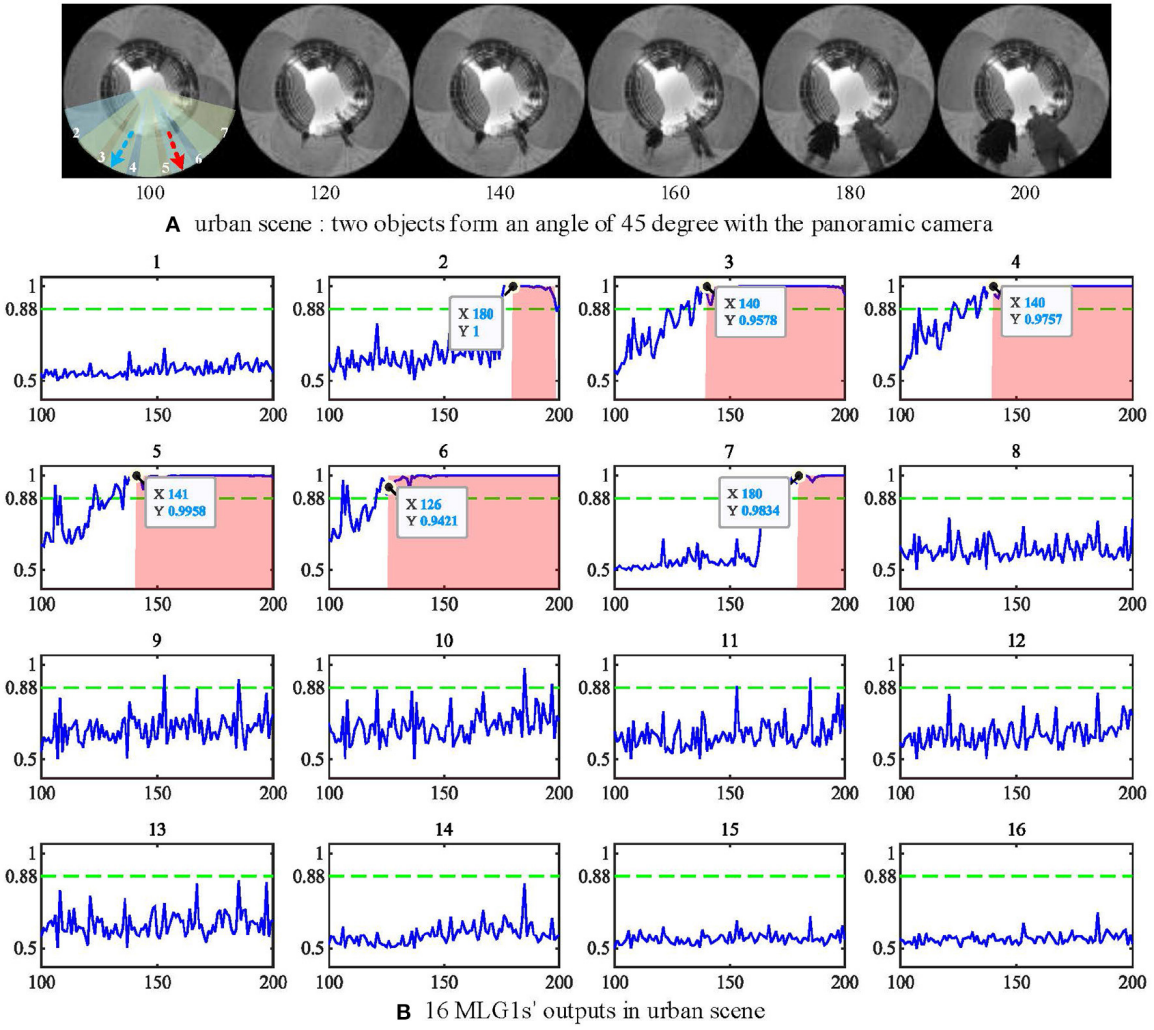
**(A)** urban scene. Two approaching people form an angle of 45 deg with the camera. The red and blue dashed lines represent the two approaching trajectories. **(B)** the outputs of 16 MLG1s in urban scene. The person at segment 6 (red line) approaches a little faster, so the neuron response first exceeds the threshold at frame 120, and the first collision warning is generated at frame 126. The Y-axis denotes a normalized membrane potential and the X-axis denotes the number of frames.

All experimental videos above subsections are recorded in the lab with a static scene. It is also interesting to test if the proposed model works for urban scenarios with uncontrolled backgrounds and moving objects. In this subsection, we will use the same panoramic camera to record outdoor videos in a city setting and investigate MLG1s model performance against a dynamic cluttered background. The urban video is recorded in a city square with a complex background containing buildings, trees, distant pedestrians, and two people approaching from different spatial locations at the same time (see Figure 13A). As shown in the figure, the two people approach the camera courses at an angle of 45 deg between each other. Their approaching trajectories are indicated in a red and a blue arrow, respectively. The experimental video can be found on Github.

When challenged with the urban scene, the parameters to cope with the noise caused by the dynamic background have been adjusted. The threshold has been set to *Ts* = 0.88 in Equation (19) and the *n*_*sp*_ = 6 in equation (20). The experimental results in Figure 13B show the response of the *MLG*1_6_ neuron first exceed the threshold at 120th frame and triggers the collision warning at 126th frame. That is because the person in segment 6 walks a little faster (see video on Github). All the locations of the looming objects in this video are perceived successfully by our proposed MLG1s model. The results demonstrate that the MLG1s model can cope with the dynamic and cluttered background effectively and robustly.

## 5. Further Discussion

The systematic off-line experiments illustrate that our proposed MLG1s model, comprising the parallel 16 partial neural networks and spike frequency adaptation mechanism, shows characteristics similar to the biological characteristics of real MLG1 neurons in the crab *Neohelice granulata*, as in Figure 2. Compared with the looming sensitive neurons LGMD1, MLG1 neurons have many similar features. For example, the both responses of neurons to looming stimuli are strongly tuned by the stimulus velocity and size. Despite these similarities, MLG1 and LGMD1 neurons differ in many aspects. The most obvious one is that the locust has only one LGMD1 neuron to cover the entire view of the eye, but the crab *Neohelice granulata* has 16 MLG1 neurons. Furthermore, the firing peak of each MLG1 neuron is influenced by the size of the receptive field. Also, the escape response to the looming stimulus in the crab *Neohelice granulata* has been proven to be highly directional. The finely tuned directional control can be accomplished suitably with a neuronal ensemble, which comprises the set of 16 MLG1 neurons.

In this paper, we have presented a plausible, bio-inspired model that can perceive the spatial location of looming stimulus. It's worth pointing out that the angular looming objects could activate neighboring MLG1 neurons in different timing, which is similar to those looming objects in direct collision course but with different sequences. As shown in Figure 6, when the looming angle is 5 deg deviation from the directly looming, the approaching object travels through segment 3, segment 2, and finally, segment 1. The relevant neurons are also activated in this sequence. If the approaching object is with a large deviation, e.g., 20 deg from a direct collision course, no MLG1s will be triggered. This is a suitable feature for a robotics vision system that could respond to relevant approaching objects but not by irrelevant ones. Further research might be required to investigate the coordination and interactions between neighboring MLG1s to understand how they could cope with diverse approaching objects. This may enable us to improve the collision selectivity of the model in the future. Moreover, the spike timing of each activated MLG1 neuron can also be encoded and extracted cues to perceive global motions around all receptive fields, which is our future work.

Except for the parallel partial neural networks, the SFA mechanism in the proposed MLG1s model demonstrates considerable preference in looming stimulus instead of receding events. Our phased results also indicate that the SFA mechanism can differentially enhance signals produced in response to different looming stimuli. However, the selectivity of the SFA mechanism has not been explored quantitatively in this paper. In the future, we will explore further the potential of the SFA mechanism and aim to embed our model into the microrobot Colias to evaluate its potential under a variety of real-world conditions.

In addition, our contrast test shows that the contrast difference between the target and background could influence the MLG1's response. This is because we have not implemented pre-processing algorithms to deal with contrast's differences. In crab, however, it can process polarization and intensity information independently and in parallel, and the final response depends on which one is most significant (Smithers et al., 2019). It is a common feature for most motion-sensitive neurons that the movement-elicited response is independent of the contrast between the background and moving stimulus (Wiersma, 1982; de Astrada and Tomsic, 2002). In the future, the relevant bio-plausible visual pre-processing methods could be implemented pre-synaptically to the MLG1s computational model to accommodate the contrast invariance.

The final experiment for the urban scene also demonstrates the robustness and effectiveness of our proposed model. However, our MLG1s model, similar to all other visual neural models, has limitations to realistic environmental conditions. The proposed model is a luminance-change-based method, it is highly dependent on lighting conditions. For example, a shadowing road with bright sunlight could be a tough challenge. Moreover, the size and distance of a looming target also influence the neural response. Similar to the crab's MLG1 neurons, the tiny target needs to be much closer to the panoramic camera to activate the MLG1s model. The MLG1s model response distance to the looming stimulus is approximately 0.3 m to a ball (0.05 m height) and 4 m to a person (1.8 m height). As a result, our MLG1s model can only perceive the motions (include looming and translating) happening within this distance and ignores long-distance motions.

## 6. Conclusions

In this paper, we have presented a visual neural network model based on biological MLG1 looming sensitive neurons in the visual nervous system of the crab *Neohelice granulata*. The MLG1s ensemble could encode the spatial location of a looming stimulus. Our proposed MLG1s model not only detects the looming spatial location but, like its biological counterpart, is sensitive to speed and size.

Furthermore, our systematic experiments demonstrated that the proposed MLG1s visual computational model works robustly and effectively. The MLG1s model may be a good candidate for visual neuromorphic sensors to perceive looming spatial location when applied in microrobots due to its low energy cost, efficiency, and reliability. In the future, we will further model the post-synaptic neural networks of MLG1 neurons. We will also investigate the potential to integrate multiple motion-sensitive neural networks to cope with complex visual stimuli for robots.

## Data Availability Statement

The datasets presented in this study can be found in online repositories. The names of the repository/repositories and accession number(s) can be found at: https://github.com/HaoLuan/BIO-INSPIRED-MODEL.

## Author Contributions

HL main author of the manuscript and developer of the software. SC and SY ideas of the project and theoretical methods. YZ, QF, and MH experiments and part of manuscript writing. All authors contributed to the article and approved the submitted version.

## Funding

This research is supported in part by National Natural Science Foundation of China under grant 62020106004, 92048301, and 12031003, the EU HORIZON 2020 projects, STEP2DYNA, European Union under grant 691154 and ULTRACEPT, European Union under grant 778062, and China Postdoctoral Science Foundation Grant 2020M682651. HL would like to thank the sponsorship of the China Scholarship Council for funding his research at University of Lincoln.

## Conflict of Interest

The authors declare that the research was conducted in the absence of any commercial or financial relationships that could be construed as a potential conflict of interest.

## Publisher's Note

All claims expressed in this article are solely those of the authors and do not necessarily represent those of their affiliated organizations, or those of the publisher, the editors and the reviewers. Any product that may be evaluated in this article, or claim that may be made by its manufacturer, is not guaranteed or endorsed by the publisher.
