# Supplementary material for: A Looming Spatial Localization Neural Network Inspired by MLG1 Neurons in the Crab Neohelice
Source: Front Neurosci. 2022 Jan 21;15:787256. doi: 10.3389/fnins.2021.787256 (PMC8814358; doi:10.3389/fnins.2021.787256)
Supplement: Supplementary file 1 [file Data_Sheet_1.PDF]

Supplementary Material

1 PANORAMIC CAMERA CALIBRATION EXPERIMENT

The panoramic camera used in the manuscript, insta360 ONE X, is a closed-source camera, and it doesn't provide lens distortion parameters. Within this section, we simply calibrate the panoramic camera to display the distortion. Fig. S1(a) is the mapping schematic diagram. The incident point P form a vertical incident angle θ with the optical axis. The red circle in figure S1(b) represents the set of all points with a vertical incidence angle of θ. The horizontal azimuth is indicated by yellow dashed lines in Fig. S1(a) and (b), respectively.

Fig. S2(a) shows that the panoramic camera is fixed on the table, and the calibration pattern is 100mm in front of the camera. The incident ray from the calibration point P forms an incident angle θ concerning the optical axis. Then, the point P has been projected into the panoramic image at a distance Γ from the image centre (see Fig. S2(c)). Fig. S2(b) shows the relationships between the distances to centre Γ versus incidence angles θ. The four calibration points are marked in four colours, respectively, in Fig. S2(c). The distances to centre Γ are measured in the image resolution of 720*720. The yellow calibration point forms an incident angle $\theta_3 = 0$, and its distance $\Gamma_3 = 185$ is around half of the image radius (i.e. 360 pixels). From the calibration angles $\theta_2 = 45$ and $\theta_4 = -45$, we can find their deviations from P3 (yellow point) are not equal. This demonstrates that the panoramic image has spatial amplification below the optical axis and spatial compression above the optical axis. In sum, the incident angle θ is inversely proportional to the distances Γ and proportional to the spatial compression ratio. Because of the panoramic camera's distortion, our proposed MLG1s model could hardly handle the approaching stimulus from a high elevation. From our point of view, the MLG1s model, as a kind of looming spatial localization neural network, is difficult to perceive the looming objects which have been spatial compressed. In crab *Neohelice granulata*, another motion-sensitive neuron, BLG1, appears to have some sensitivity to stimulus elevation. This inspires us to integrate more neuronal computational models to extract and encode motion cues to address the challenges in realistic scenes.

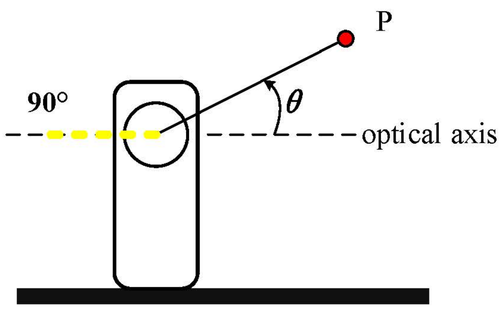

(a)

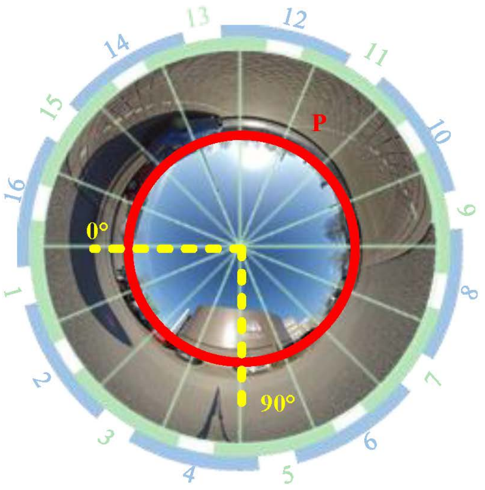

(b)

Figure S1. The mapping relationship between realistic scene to the panoramic image.

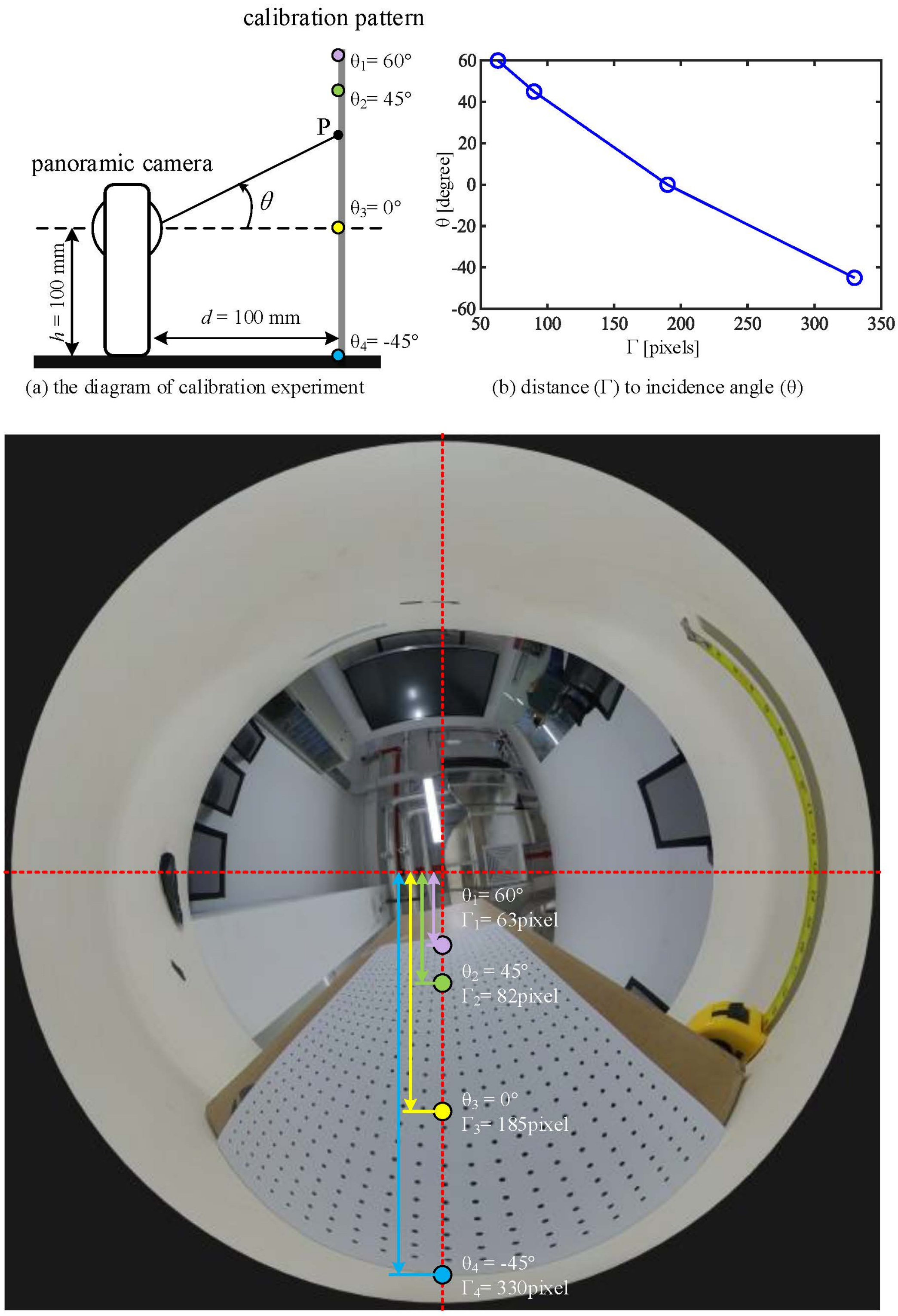

(c) calibration point

Figure S2. (a) The diagram of calibration experiment. (b) Relationship between the distance to centre Γ versus incidence angle θ. (c) The calibration pattern. The distances to centre Γ are measured in the image resolution of 720*720.

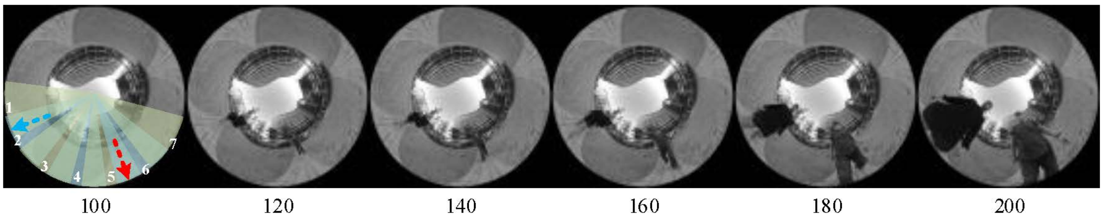

(a) urban scene 1: two objects form an angle of 90 degree with the panoramic camera

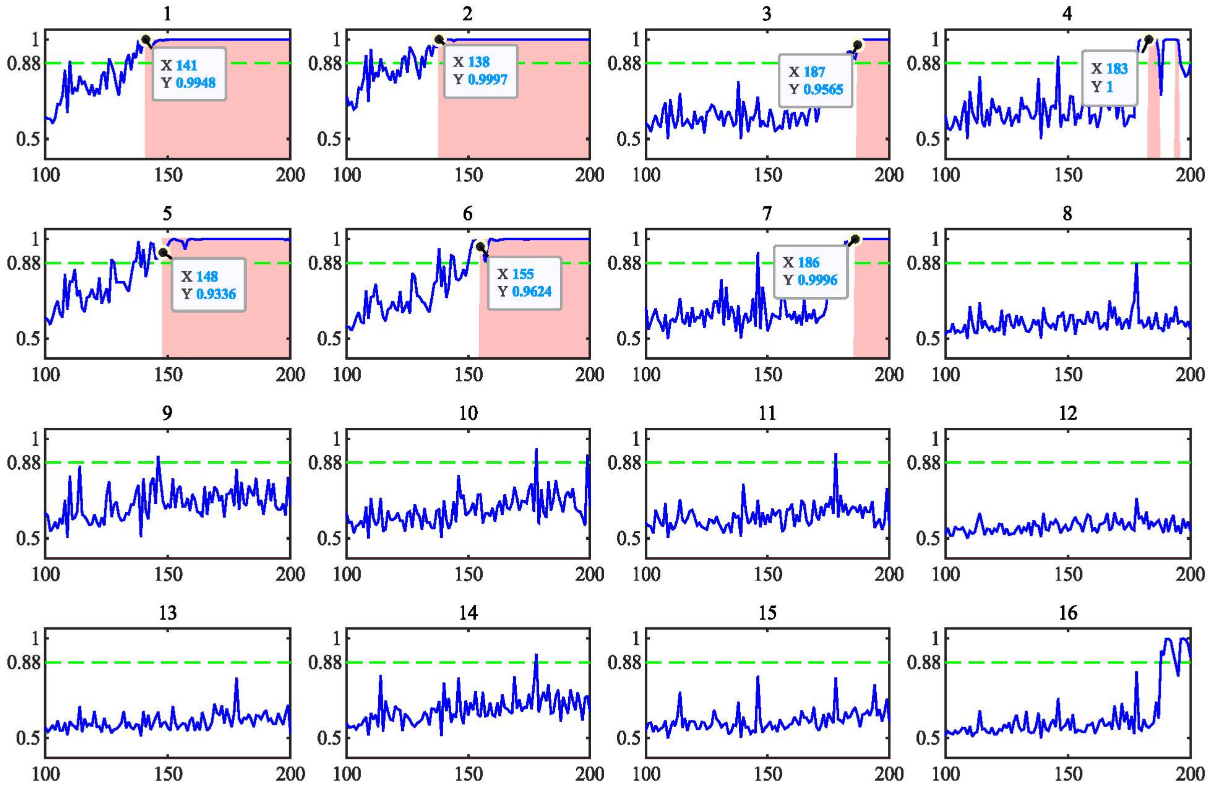

(b) 16 MLG1s' outputs in urban scene 1

Figure S3. (a) urban scene 1. Two approaching people form an angle of 90 deg with the camera. The red and blue dashed lines represent the two approaching trajectories. (b) the outputs of 16 MLG1s in urban scene 1. The people at segment 2 (blue line) approaches from a closer distance, so the corresponding output first exceed the threshold and then the first collision warning generated at 138th frame in this test.

2 REALISTIC URBAN SCENE EXPERIMENTS

We here present our proposed MLG1s model performances in realistic urban scenes. As in the manuscript, we adjust the parameters $Ts = 0.88$ and the $n_{sp} = 6$. In these urban scene experiments, two people approach the panoramic camera with angles of 90 deg (Fig.S3) and 180 deg (Fig.S4), respectively. The model outputs show that all MLG1 neuronal responses correspond to the looming motions of the relevant segments. Although the rest of the neurons are affected by the noises, they haven't been activated successfully. Thus, these experiments demonstrate the robustness and effectiveness of the proposed MLG1s model against the realistic scenes. The experimental videos can be found on `https://github.com/HaoLuan/BIO-INSPIRED-MODEL`.

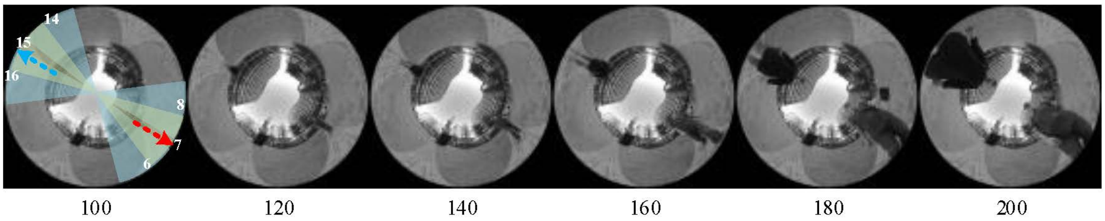

(a) urban scene 2: two objects form an angle of 180 degree with the panoramic camera

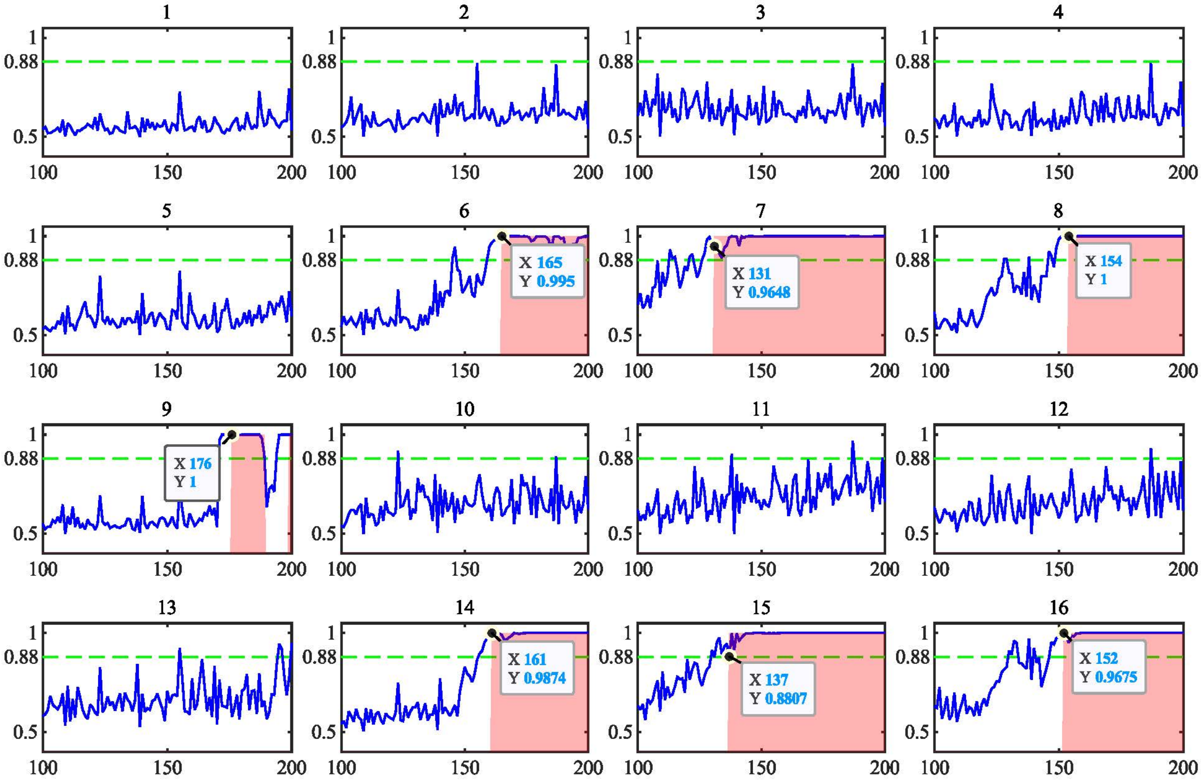

(b) 16 MLG1s' outputs in urban scene 2

Figure S4. (a) urban scene 2. Two approaching people form an angle of 180 deg with the camera. The red and blue dashed lines are the two approaching trajectories. (b) the outputs of 16 MLG1s in urban scene 2. In this test, two people approaching at the same speed and distant, thus the relevant segments' outputs exceed the threshold at almost the same time (i.e. 131st frame in segment 7 and 137th frame in segment 15). The rest neurons haven't been activated.
